# Prevention of oral mucositis in patients undergoing cancer chemotherapy using betamethasone mouthwash: A multicenter randomized controlled trial protocol

**DOI:** 10.1371/journal.pone.0345991

**Published:** 2026-04-08

**Authors:** Sakiko Soutome, Tomofumi Hamada, Hiromi Nishi, Eiji Iwata, Hirokazu Nakahara, Toshiro Yamamoto, Tatsushi Matsumura, Masaya Nishikawa, Kei Tomihara, Koichiro Ueki, Mamoru Noda, Makoto Shimanishi, Madoka Funahara, Masami Kawahara, Yoshiko Yamamura, Shinpei Matsuda, Kensuke Yoshida, Sanshiro Kanazawa, Minako Otsuji, Nagato Natsume, Masahiro Umeda, Research Committee of the Japanese Society of Oral Care

**Affiliations:** 1 Department of Oral Health, Nagasaki University Graduate School of Biomedical Sciences, Nagasaki, Japan; 2 Center for Clinical and Translational Research, Toyama University Hospital, Toyama, Japan; 3 Department of Oral and Maxillofacial Surgery, Social Medical Corporation Hakuaikai Sagara Hospital, Kagoshima, Japan; 4 Department of General Dentistry, Hiroshima University Hospital, Hiroshima, Japan; 5 Department of Oral and Maxillofacial Surgery, Kakogawa Central City Hospital, Hyogo, Japan; 6 Department of Dentistry and Oral Surgery, Osaka Metropolitan University Hospital, Osaka, Japan; 7 Department of Dental Medicine, Graduate School of Medical Science, Kyoto Prefectural University of Medicine, Kyoto, Japan; 8 Department of Oral and Maxillofacial Surgery, Wakayama Medical University, Wakayama, Japan; 9 Department of Dentistry and Oral Surgery, Nishichita General Hospital, Aichi, Japan; 10 Division of Oral and Maxillofacial Surgery, Niigata University Graduate School of Medical and Dental Science, Niigata, Japan; 11 Department of Oral and Maxillofacial Surgery, Division of Medicine, Interdisciplinary Graduate School, University of Yamanashi, Yamanashi, Japan; 12 Department of Endodontics, School of Dentistry, Faculties and Graduate Schools, Iwate Medical University, Iwate, Japan; 13 Department of Dental and Oral Surgery, Oji General Hospital, Hokkaido, Japan; 14 School of Oral Health Sciences Faculty of Dentistry, Kyushu Dental University, Fukuoka, Japan; 15 Laboratory of Clinical Pharmacy, School of Pharmacy, Aichi Gakuin University, Aichi, Japan; 16 Department of Oral and Maxillofacial Surgery, Faculty of Medicine, Juntendo University, Tokyo, Japan; 17 Department of Dentistry and Oral Surgery, Unit of Sensory and Locomotor Medicine, Division of Medicine, Faculty of Medical Sciences, University of Fukui, Fukui, Japan; 18 Department of Drug Safety and Risk Management, School of Pharmacy, Tokyo University of Pharmacy and Life Sciences, Tokyo, Japan; 19 Department of Oral and Maxillofacial Surgery, Graduate School of Medicine, The University of Tokyo, Tokyo, Japan; 20 Dentistry/Oral and Maxillofacial Surgery, Kagoshima City Hospital, Kagoshima, Japan; 21 Division of Research and Treatment for Oral Maxillofacial Congenital Anomalies, School of Dentistry, Aichi Gakuin University, Aichi, Japan; University of Insubria, ITALY

## Abstract

**Background:**

Oral mucositis is a frequent adverse effect of chemotherapy that significantly affects patients’ quality of life and treatment continuity. Despite its clinical relevance, effective preventive strategies remain limited. This study aims to compare the efficacy and safety of betamethasone mouthwash with those of sodium gualenate hydrate mouthwash for preventing chemotherapy-induced oral mucositis.

**Methods:**

This Phase II, multicenter, open-label, randomized controlled trial aims to enroll 296 adult patients (≥18 years) undergoing chemotherapy for solid tumors (excluding head and neck and hematological cancers). Participants will be randomized 1:1 into two arms: a betamethasone mouthwash (intervention) group and a sodium gualenate hydrate mouthwash (control) group. The primary outcome is the prevention of Grade 1 oral mucositis, assessed using the Common Terminology Criteria for Adverse Events v3.0 and v5.0. Secondary outcomes include prevention of Grade 2–3 oral mucositis, the incidence of oral candidiasis, and chemotherapy completion rates. Data will be analyzed using Kaplan–Meier survival curves, log-rank tests, and Cox regression models.

**Expected Outcomes:**

This study aims to establish betamethasone mouthwash as an effective preventive strategy for potentially enhancing the quality of life of patients with cancer.

**Trial registration:**

This study was registered with the Japan Registry of Clinical Trials on September 20, 2024 (jRCTs071240060).

## Introduction

Chemotherapy frequently causes oral mucositis, a painful and debilitating condition that can impair oral intake, worsen nutritional status, and lead to treatment modification or interruption [1,2]. Despite various suggested preventive approaches, including cryotherapy, oral care, analgesics, and zinc supplementation [3], high-quality evidence supporting their efficacy remains limited, and current management is largely symptomatic.

Topical corticosteroids are considered promising because of their potent anti-inflammatory effects. A protocol for a multicenter randomized controlled trial evaluating betamethasone valerate ointment for radiation-induced oral mucositis (the Bet-ROM study) has been published by our group [4], providing important methodological insight. Although several recent studies have shown that steroid-containing mouthwashes can reduce mucositis associated with targeted therapy or chemotherapy in breast cancer [5–7], evidence supporting their use for chemotherapy-induced oral mucositis in patients with other solid tumors remains insufficient. Furthermore, steroid-containing mouthwashes are not commercially available in Japan, and nonsteroidal agents commonly used in practice lack robust evidence.

Given the need for an effective prophylactic strategy applicable to a broader chemotherapy population, we designed a randomized controlled trial to evaluate the safety and preventive efficacy of betamethasone mouthwash in patients undergoing chemotherapy for solid tumors.

## Materials and methods

This study was designed to verify that betamethasone mouthwash inhibits severe oral mucositis.

This study was approved by the Clinical Research Review Board of Nagasaki University (No. CRB7180001) and registered in the Japan Registry of Clinical Trials (jRCT) on September 20, 2024 (jRCTs071240060). The details are available at https://jrct.niph.go.jp/re/reports/detail/84186. Any protocol changes that impact the study conduct and/or participant risk–benefit profile, including changes in objectives, design, sample size, participant characteristics, staff changes, or significant administrative aspects, will require approval from the relevant Institutional Review Board. Minor protocol corrections and/or clarifications that do not affect the study design or the participant risk–benefit profile are viewed as administrative changes and will be documented internally. The study investigators will have full access to and ownership of all the data. De-identified data will be made available to other interested investigators for additional analyses upon reasonable request and with an appropriate data use agreement after reporting the primary outcomes. The findings of this study will be disseminated through scientific and professional conferences, and peer-reviewed journals.

### Study design

This multicenter, open-label, randomized, controlled, Phase II trial with two parallel arms will follow the CONSORT guidelines and adhere to the ethical principles outlined in the Declaration of Helsinki.

### Endpoints

*Primary Outcome measure*: Incidence of Grade 1 oral mucositis during an 8-week observation period.

*Secondary Outcome measures*: Grades 2–3 mucositis, incidence of oral candidiasis, and chemotherapy completion rates.

Grade 4 mucositis is not included because it is extremely rare, often requires urgent medical intervention, and cannot be ethically or practically evaluated in the outpatient setting targeted by this study.

### Eligibility criteria

*Inclusion Criteria*: Adults (≥18 years) undergoing chemotherapy for solid tumors (excluding head and neck and hematologic cancers) who provide written informed consent.

*Exclusion Criteria*: History of hypersensitivity to betamethasone, existing oral mucositis or oral candidiasis, and the inability to perform gargling.

### Randomization

Random allocation will assign patients in a 1:1 ratio to the betamethasone and sodium gualenate hydrate mouthwash groups.

### Treatment and assessment schedule

The study period is 8 weeks from the chemotherapy cycle.

*Betamethasone (Intervention) group:* Before or after the start of chemotherapy, in addition to the usual oral management, patients will mouthwash with 10 mL of 0.01% betamethasone solution (Linolosal® Injection 20 mg (0.4%) (Wakamoto Pharmaceutical Co., Ltd., Tokyo) contains 5 mL of 26.3 mg of betamethasone sodium phosphate and 20 mg of betamethasone), adjusted to a total volume of 200 mL with tap water.) for 30 s to 1 min four times a day (after each meal and before bedtime) for 56 days.

*Sodium gualenate hydrate (Control) Group:* Before or after the start of chemotherapy, in addition to the usual oral management, patients will mouthwash with 10 mL of 4% sodium gualenate hydrate mouthwash (Azunol ® Gargle liquid, ROHTO NITTEN Co., Ltd contains sodium azulene sulfonate hydrate 40 mg in 1 mL; dissolve 4–6 mg (one squeeze or 5–7 drops) in an appropriate amount of approximately 100 mL water according to attached document) for 30 s to 1 min four times a day (after each meal and before bedtime) for 56 days.

Even if no food is consumed, the patient will be instructed to rinse the mouth at appropriate mealtimes. The use of steroid ointments in the oral cavity during the study period shall be prohibited in both groups. This study does not require any mandatory concomitant medications or therapies; however, concomitant drugs or therapies shall not be restricted.

All patients received standardized instructions for specific oral care involves instructing patients to use a toothbrush, by scrubbing or bass method, and interdental brushes and dental floss to remove dental plaque, basically three times a day so that patients can effectively perform self-care themselves. After instruction, the dentist or dental hygienist will perform professional mechanical tooth cleaning using oral hygiene tools every 1–2 weeks. The tongue and oral mucosa will be cleaned using a sponge brush and moisturizing gel. This unified protocol is applied across all participating centers to minimize variability. Regular oral management shall include modifying the form of food, administering non-steroidal anti-inflammatory drugs (NSAIDs) or opioids for pain management, providing oral care, and using moisturizers.

**Fig 1:**
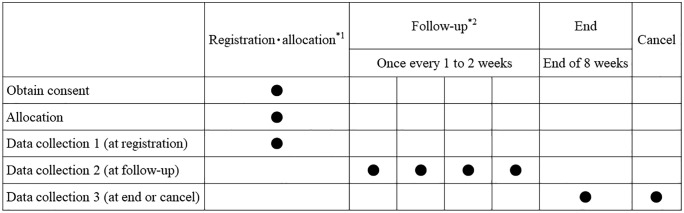
SPIRIT schedule of enrollment, interventions.

*Data collection:* The data collection schedule is shown in Fig 1.

*2 For inpatient treatment, the procedures should be performed weekly or at the time of admission. For outpatient treatment, the procedures should be performed on the day of therapy. In the case of treatment cycle delay, gargling and information collection should be adjusted according to the delay. Dental visits are not necessarily mandatory; it is acceptable for a dentist to visit locations, such as the outpatient chemotherapy room, for examinations.

The following data will be collected at registration 1) Background information including age, sex, medical history, prescribed medications, primary site of disease, smoking history, drinking history, and body weight; 2) Treatment-related factors including date of treatment initiation, name(s) of anticancer drug(s), concurrent medications/therapies, clinical test findings (in-hospital tests), neutrophil count, lymphocyte count, and serum albumin and creatinine levels; and 3) Oral findings including presence and grade of oral mucositis (Common Terminology Criteria for Adverse Events [CTCAE] v3.0 and v5.0), number of remaining teeth, presence of dentures, periodontal examination results (pocket depth, bleeding, and mobility), plaque adherence level (oral hygiene index-simplified), denture hygiene (if wearing dentures), presence and location of untreated teeth, and extent of alveolar bone resorption in patients with available dental radiographs.

The following data will be collected during the follow-up visits after allocation 1) Presence and grade of oral mucositis (CTCAE v3.0 and v5.0), the date of onset, if applicable; 2) Presence and timing of onset of oral candidiasis; and 3) Use of the investigational drug and adverse events related to the investigational drug including inflammatory changes such as redness and swelling in the oral mucosa, taste disturbances, oral infections, and other adverse events.

The following data will be collected at the end of or upon discontinuation of the study: 1) Treatment-related factors including start and end dates of chemotherapy; completion status of chemotherapy; use of NSAIDs local anesthetics (mouthwashes, gels, etc.), or opioids; the lowest neutrophil and lymphocyte counts during treatment; the lowest serum albumin level during treatment; the highest serum creatinine level during treatment; use and timing of granulocyte-colony stimulating factor (G-CSF) preparations, and prognostic Nutritional Index (PNI); 2) Oral mucositis grade (CTCAE v3.0 and v5.0) and date of onset; 3) Incidence and date of onset of oral candidiasis; 4) Investigational drug usage status; and 5) Adverse events related to the investigational drug including inflammatory changes such as redness and swelling of the oral mucosa, taste disturbances, oral infections, and other adverse events.

### Monitoring and management for oral candidiasis

All participants will be monitored for oral candidiasis at each visit during the 8-week study period. Professional oral examinations by dentists or dental hygienists will be performed every 1–2 weeks to facilitate early detection of fungal infections. Participants will also be instructed to report any new oral symptoms (e.g., burning sensation, dysgeusia, white plaques) between scheduled visits. Oral candidiasis will be diagnosed by an oncologist or dentist based on the presence of characteristic clinical findings, including removable white plaques, erythematous areas, angular cheilitis, or pseudomembranous lesions. Microbiological culture will be performed at the discretion of the treating clinician; however, diagnosis will rely on clinical assessment regardless of culture results, consistent with established clinical practice.

If oral candidiasis is suspected or confirmed, antifungal therapy will be initiated promptly. Acceptable treatments include amphotericin B suspension, miconazole oral gel, or miconazole buccal tablets. In the betamethasone group, steroid mouthwash will be temporarily suspended upon diagnosis of candidiasis and resumed after clinical resolution. In the control group, sodium gualenate hydrate mouthwash will be continued without interruption. All cases of candidiasis, treatment initiation dates, antifungal duration, and outcomes will be recorded in the case report form.

### Patient assignment and data management

Patients will be recruited from Nagasaki University Hospital, Sagara Hospital, Hiroshima University Hospital, Kakogawa Central City Hospital, Osaka Metropolitan University Hospital, University Hospital Kyoto Prefectural University of Medicine, Wakayama Medical University Hospital, Nishichita General Hospital, Niigata University Medical and Dental Hospital, University of Yamanashi Hospital, Iwate Medical University Hospital, and Oji General Hospital (South to North). Patient recruitment began on September 20, 2024, and is planned to continue until December 31, 2026.

**Fig 2:**
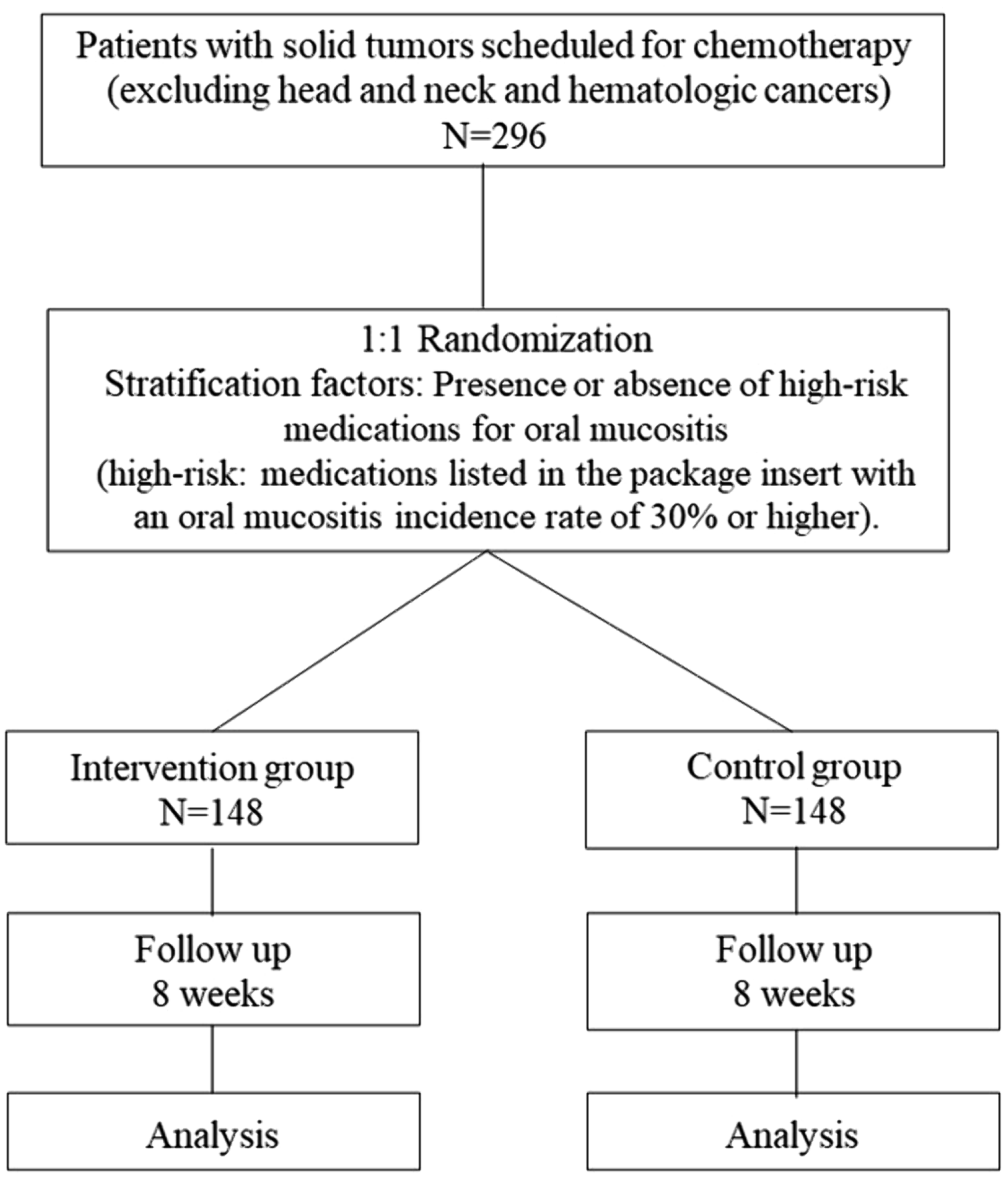
Flowchart of treatment for mouthwash patients.

The Clinical Research Center of Kyushu Dental University will randomly allocate patients after the incidence of Grade 1 oral mucositis to the intervention or control group in a 1:1 ratio using a stratified allocation method that minimizes the effects of allocation adjustment factors. The allocation algorithm will be determined by the person responsible for the biostatistical analysis based on the presence of high-risk medications for oral mucositis (high-risk: medications with an oral mucositis incidence rate ≥30% listed in the package insert). Data management will be performed at Kyushu Dental University (M.F.). (Fig 2)

This study will use paper-based case report forms (CRFs) for data collection. The principal investigator or coinvestigator at each participating site will prepare the CRF for each participant. The data required according to the protocol (version1.1, August 4, 2024) will be recorded in the CRF. When making changes or corrections, the principal investigator or sub-investigator at the participating site will, without using a correction fluid or a similar tool, cross out the incorrect entry with a double line, affix their seal or signature next to the corrected entry, and note the correction date. The CRF signed by the principal investigator at the participating site will be treated as the original, and the principal investigator will verify that all recorded data are complete and accurate. During the observation period, a copy of the completed CRF will be submitted to MF at Kyushu Dental University. If any changes or corrections are made, the original CRF shall be corrected as described above, and an updated copy shall be forwarded to MF at Kyushu Dental University. After the observation period, the principal investigator at each participating facility shall make copies of all CRFs and submit the originals to MF at Kyushu Dental University by mail or in person; copies of CRFs will be stored at each participating facility during the retention period specified in the research protocol.

### Sample size calculation

In a previous study by Kuba et al., steroid mouthwashes reduce the risk of oral mucositis by 27% (0.68 times). Assuming an incidence rate of oral mucositis during chemotherapy for malignant tumors is 40%, and for between-group comparisons, Fisher’s exact probability test is used. With a two-sided test α error of 0.2, a power of 0.8, the required sample size is 266 cases (133 cases per group). Considering 10% dropout rate, the target number of administration cases is set at 296 cases (148 cases per group).

### Study period

The study period of this trial will be from the day it is released by the Japan Registry of Clinical Trials (jRCT) on September 20, 2024 to December 31, 2026; the participant entry period will be from the day it is released by jRCT on September 20, 2024 to March 31, 2026; Data collection and CRF will be enrolled from September 20, 2024 to June 30, 2026.

### Statistical analysis

*Main analysis and assessment criteria*: The incidence rates of Grade 1 oral mucositis in each group will be calculated. The differences in incidence rates (risk difference), ratios of incidence rates (risk ratio), and their 95% confidence intervals will be calculated. Fisher’s exact test at a two-sided significance level of 5% will be used to test the superiority of the steroid mouthwash group along with the incidence rate of oral mucositis in each group. Estimate the cumulative incidence rate at each time point using the Kaplan-Meier method with a log-rank test for reference, and the hazard ratio (HR) and a 95% confidence interval are analyzed.

*Secondary analysis:*

1) A summary of each endpoint will be presented.
2) The incidence rates of Grade 2 oral mucositis in each group will be calculated. The differences in incidence rates (risk difference), ratios of incidence rates (risk ratio), and their 95% confidence intervals will be calculated. Fisher’s exact test at a two-sided significance level of 5% will be used to test the superiority of the steroid mouthwash group along with the incidence rate of oral mucositis in each group. Estimate the cumulative incidence rate at each time point using the Kaplan-Meier method with a log-rank test for reference, and the hazard ratio (HR) and a 95% confidence interval are analyzed.
3) The Kaplan–Meier method will be used to calculate the incidence of oral candidiasis in the intervention and control groups, and the differences between the two groups will be compared using the log-rank test.
4) Factors related to the incidence of oral mucositis in each grade will be evaluated using Cox regression analysis.
5) No adjustment shall be made for multiplicity.

### Interim analysis and monitoring

No interim analyses will be performed. In-house monitoring will be conducted by Nagasaki University, independent of the investigators and data center, to assess and improve study progress, data integrity, and patient safety. Monitoring will be conducted at each site during the first and final assessments, and for all confirmed discontinuations and serious illnesses.

### Safety monitoring

When a disease or condition occurs, the principal investigator or research staff at the site shall promptly initiate appropriate measures (including explanations) for the patient, such as discontinuation or suspension of the relevant clinical research, and record the event name (diagnostic name), date of onset, severity, causal relationship, predictability, outcome, and date of determination of outcome. If the disease or condition continues at the time of the final observation, the investigator or investigator in charge of the study at the site shall continue the follow-up observation until the disease or condition recovers to its original state or becomes clinically stable. The principal investigator will collate the diseases or conditions reported by the investigators from each institution and report them to the Clinical Research Review Board at Nagasaki University.

### Auditing

No audit will be conducted.

## Discussion

Previous studies have suggested the potential usefulness of topical corticosteroids for preventing therapy-related oral mucositis. Early reports from Japan in the 1980s indicated that steroid ointments were effective for radiation-induced mucositis [8], although concerns regarding the development of oral candidiasis limited their widespread adoption. However, a multicenter observational study later demonstrated that the major risk factors for oral candidiasis during radiotherapy were leukopenia and the severity of mucositis, and that steroid application itself did not increase the risk; rather, the incidence tended to decrease [9]. Furthermore, a scoping review reported recently by Funahara et al. described that topical corticosteroid therapy, particularly dexamethasone-based mouthwashes and ointments, was associated with significant reductions in oral mucositis incidence and severity, with no adverse events such as oral candidiasis reported [10].

Our group previously conducted a pilot study suggesting that betamethasone valerate ointment may prevent severe mucositis in patients undergoing chemoradiation or bioradiation therapy for oral and oropharyngeal cancer, which led to the development of the Bet-ROM multicenter randomized trial [4]. In addition, several phase III studies have reported that steroid ointments or steroid-containing mouthwashes reduced the incidence of oral mucositis associated with everolimus or chemotherapy in patients with breast cancer [6,11]. These findings support the concept that topical corticosteroids may have a broader role in the prevention of therapy-related mucositis; however, evidence has been largely limited to specific cancer types or targeted agents. Furthermore, in Japan, steroid-containing mouthwashes are not commercially available or reimbursed by the National Insurance system, and nonsteroidal mouthwashes such as sodium gualenate hydrate are often used despite limited evidence. The present trial was therefore designed to address this clinical gap by investigating the preventive efficacy and safety of a betamethasone-based mouthwash in patients receiving chemotherapy for solid tumors.

The use of a corticosteroid-based intervention, such as betamethasone, is grounded in evidence suggesting that its anti-inflammatory properties can mitigate mucosal damage [2]. By comparing betamethasone mouthwash with sodium gualenate hydrate mouthwash, a widely used but less targeted treatment, this trial seeks to provide definitive guidelines for clinical practice. Our findings may have several implications. If betamethasone mouthwash is proven effective, it may fill a critical gap in current mucositis management protocols. Because existing strategies primarily focus on symptomatic relief rather than prevention, this approach aligns with the proactive care model. Importantly, the study design, leveraging a randomized multicenter approach, ensures that the results will be applicable across diverse clinical settings, enhancing their generalizability within the specified patient population. Additionally, our trial will address the notable clinical challenge of balancing efficacy with safety.

Concerns regarding the use of topical corticosteroids in the oral cavity, particularly their association with oral candidiasis, are well-documented. The inclusion of oral candidiasis as a secondary endpoint allows for a nuanced understanding of the risk–benefit profile of the intervention. Early results from prior observational studies suggested that the benefits of corticosteroid use outweigh these risks when carefully monitored [10], a hypothesis that we aim to confirm through rigorous data collection and analysis. Furthermore, structured data collection on adverse events and quality indicators, such as chemotherapy completion rates, will shed light on the broader impact of oral mucositis prevention. Betamethasone mouthwash can indirectly support uninterrupted chemotherapy schedules by improving oral health outcomes and potentially improving the overall efficacy of cancer treatment. The findings of this study could catalyze further research on localized steroid treatment for oral complications in the field of oncology. This trial represents a critical step toward redefining oral mucositis management and offers hope for improved patient experience and outcomes during chemotherapy.

This study has some limitations. First, the open-label nature of the trial could introduce performance and detection biases, because participants and clinicians are aware of the group assignments. This may influence subjective outcomes such as the grading of mucositis severity. Second, the exclusion of patients with head and neck or hematological malignancies limits the generalizability of the findings, as these populations often have a higher risk of oral mucositis. Additionally, the lack of intermediate endpoints such as quality-of-life measures may restrict insights into the broader impact of the intervention. Finally, the study does not include a placebo group, which may provide additional context for evaluating the efficacy of both the intervention and control groups. Future investigations should explore other corticosteroid formulations or combinations with adjunct therapies to optimize efficacy and patient adherence. Additionally, examining quality-of-life metrics at greater depths would provide a more holistic view of treatment benefits.

If successful, the findings of this trial have the potential to establish betamethasone mouthwash as an effective and targeted intervention, offering a proactive approach to mitigate chemotherapy-related complications. Additionally, the insights gained from this study may pave the way for broader applications of corticosteroid-based mouthwash in oncology care and inspire further research to refine preventive and therapeutic options for oral health in patients with cancer.

This trial represents a critical advancement in supportive cancer care aimed at enhancing patient outcomes and overall treatment success rates by addressing the common and burdensome side effects of chemotherapy.

## Supporting information

S1 File SPIRIT checklist. (DOC)

S2 File Study Protocol (Japanese). (DOCX)

S3 File Study protocol (Translated English). (DOCX)

S4 File Membership of the Research Committee. (DOCX)
