# Supplementary material for: Prevention of oral mucositis in patients undergoing cancer chemotherapy using betamethasone mouthwash: A multicenter randomized controlled trial protocol
Source: PLoS One. 2026 Apr 8;21(4):e0345991. doi: 10.1371/journal.pone.0345991 (PMC13061232; doi:10.1371/journal.pone.0345991)
Supplement: S2 File — (DOCX) [file pone.0345991.s002.docx]

**ベタメタゾン含嗽剤の口腔粘膜炎**

**発症予防に関する多機関共同**

**ランダム化比較試験**

**研究代表医師**

**長崎大学病院**

作成日：2025年6月18日

版数：第2.1版

目次

[**0** **研究の要約** 6](#_Toc170767134)

[**1** **はじめに** 7](#_Toc170767135)

[**2** **研究の背景** 7](#_Toc170767136)

[**3** **研究の目的・意義** 8](#_Toc170767137)

[**4** **研究の概要** 8](#_Toc170767138)

[4.1 研究の方法 8](#_Toc170767139)

[4.2 被験薬／被験機器 8](#_Toc170767140)

[4.3 被験薬の管理 11](#_Toc170767141)

[4.4 研究対象者の選定方針 11](#_Toc170767142)

[4.5 治療（プロトコール治療）の概要 12](#_Toc170767143)

[4.6 治療（プロトコール治療）の基準 12](#_Toc170767144)

[4.7 併用治療 13](#_Toc170767145)

[4.8 併用禁止薬／併用禁止機器／併用禁止療法 13](#_Toc170767146)

[4.9 併用制限薬／併用制限機器／併用制限療法 13](#_Toc170767147)

[4.10 評価、収集項目とスケジュール 13](#_Toc170767148)

[⚫ スケジュール（表） 13](#_Toc170767149)

[⚫ 観察・検査項目について 13](#_Toc170767150)

[4.11 研究終了後の治療法 14](#_Toc170767151)

[4.12 研究対象者への検査結果等の提供 14](#_Toc170767152)

[4.13 個々の研究対象者における中止基準 15](#_Toc170767153)

[**5** **研究対象者に生じる予測される利益、不利益** 15](#_Toc170767154)

[5.1 予測される利益 15](#_Toc170767155)

[5.2 予測される不利益 15](#_Toc170767156)

[5.3 予測される副作用 15](#_Toc170767157)

[5.4 予測される不具合 15](#_Toc170767158)

[**6** **本研究に参加しない場合の治療法** 15](#_Toc170767159)

[**7** **目標症例数** 16](#_Toc170767160)

[**8** **研究実施期間** 16](#_Toc170767161)

[**9** **症例登録方法** 16](#_Toc170767162)

[**10** **無作為化の方法と割り付け調整因子** 17](#_Toc170767163)

[**11** **盲検化の管理** 17](#_Toc170767164)

[11.1 盲検化の方法 17](#_Toc170767165)

[11.2 開鍵（キーオープン）の必要性の判断および手順 17](#_Toc170767166)

[**12** 評価項目 17](#_Toc170767167)

[12.1 主要評価項目 17](#_Toc170767168)

[12.2 副次評価項目 17](#_Toc170767169)

[12.3 安全性評価項目 17](#_Toc170767170)

[**13** **統計解析** 18](#_Toc170767171)

[13.1 解析対象集団の特定 18](#_Toc170767172)

[13.2 主要評価項目の解析 18](#_Toc170767173)

[13.3 副次評価項目の解析 18](#_Toc170767174)

[13.4 安全性評価の解析 19](#_Toc170767175)

[13.5 部分集団解析 19](#_Toc170767176)

[13.6 中間解析計画 19](#_Toc170767177)

[13.7 欠測データの取り扱い等 19](#_Toc170767178)

[13.8 統計解析計画の変更 19](#_Toc170767179)

[**14** **データマネジメント** 19](#_Toc170767180)

[14.1 症例報告書の種類 19](#_Toc170767181)

[14.2 原資料の特定 20](#_Toc170767182)

[14.3 症例報告書に直接記入され、かつ原資料と解すべき項目 20](#_Toc170767183)

[**15** **疾病等の取り扱い** 20](#_Toc170767184)

[15.1 用語の定義 20](#_Toc170767185)

[15.2 疾病等の評価 21](#_Toc170767186)

[15.3 予測できる疾病等 21](#_Toc170767187)

[15.4 疾病等が発現した場合の研究対象者への措置 21](#_Toc170767188)

[15.5 疾病等の報告 21](#_Toc170767189)

[15.6 重篤な疾病等の報告 22](#_Toc170767190)

[15.7 不具合の報告 22](#_Toc170767191)

[**16** **効果安全性評価委員会** 22](#_Toc170767192)

[**17** **研究計画書の遵守、変更及び不適合（研究計画書からの逸脱等）** 22](#_Toc170767193)

[17.1 研究計画書の遵守 22](#_Toc170767194)

[17.2 研究計画書の変更 22](#_Toc170767195)

[17.3 不適合の管理（研究計画書からの逸脱等） 23](#_Toc170767196)

[**18** **インフォームド・コンセントについて** 23](#_Toc170767197)

[18.1 インフォームド・コンセントを受ける手続き 23](#_Toc170767198)

[18.2 研究対象者等及びその関係者からの相談等への対応 23](#_Toc170767199)

[18.3 代諾者等からインフォームド・コンセントを受ける場合 24](#_Toc170767200)

[18.4 インフォームド・アセント 24](#_Toc170767201)

[18.5 研究対象者等に対する同意取得が不要な場合 24](#_Toc170767202)

[**19** **個人情報等の取り扱い** 24](#_Toc170767203)

[19.1 個人情報の管理について 24](#_Toc170767204)

[19.2 保有個人情報のうち本人を識別することができるものの開示 24](#_Toc170767205)

[19.3 遺伝的特徴等に関する情報の取扱い（偶発的所見を含む） 25](#_Toc170767206)

[19.4 原資料等の閲覧 25](#_Toc170767207)

[**20** **情報の保管および破棄の方法** 25](#_Toc170767208)

[**21** **情報の二次利用** 26](#_Toc170767209)

[**22** **情報のバイオバンクとしての利用** 26](#_Toc170767210)

[**23** **研究の資金源等、研究に係る利益相反管理** 26](#_Toc170767211)

[23.1 研究の資金源等 26](#_Toc170767212)

[23.2 利益相反管理 26](#_Toc170767213)

[**24** **研究対象者の費用負担・謝礼について** 26](#_Toc170767214)

[**25** **健康被害に対する補償** 26](#_Toc170767215)

[**26** **定期報告** 27](#_Toc170767216)

[26.1 認定臨床研究審査委員会／管理者への定期報告 27](#_Toc170767217)

[26.2 厚生労働大臣への定期報告 27](#_Toc170767218)

[**27** **研究の終了と中止** 27](#_Toc170767219)

[27.1 研究の終了 27](#_Toc170767220)

[27.2 研究の中止 27](#_Toc170767221)

[**28** **研究の情報公開及び結果の公表** 28](#_Toc170767222)

[28.1 研究の登録 28](#_Toc170767223)

[28.2 研究結果の公表 28](#_Toc170767224)

[28.3特定臨床研究の個々の対象者の匿名化されたデータを共有 29](#_Toc170767225)

[**29** **品質管理及び品質保証** 29](#_Toc170767226)

[29.1 モニタリング 29](#_Toc170767227)

[29.2 監査 29](#_Toc170767228)

[**30** **研究成果の帰属（知的財産権）** 29](#_Toc170767229)

[**31** **研究の実施体制** 29](#_Toc170767230)

[31.1 研究代表医師 29](#_Toc170767231)

[31.2 研究事務局 30](#_Toc170767232)

[31.3 実施医療機関および研究責任医師 30](#_Toc170767233)

[31.4 データマネジメント責任者 30](#_Toc170767234)

[31.5 統計解析責任者 30](#_Toc170767235)

[31.6 モニタリングに関する責任者 30](#_Toc170767236)

[31.7 監査に関する責任者 30](#_Toc170767237)

[31.8 研究・開発計画支援担当者 30](#_Toc170767238)

[31.9 調整管理事務担当者 30](#_Toc170767239)

[31.10 研究責任医師または研究代表医師以外に研究を総括する者 30](#_Toc170767240)

[31.11 その他臨床研究に関連する臨床検査施設並びに医学的及び技術的部門・機関 31](#_Toc170767241)

[31.12 開発業務受託機関 31](#_Toc170767242)

[**32** 文献 31](#_Toc170767243)

[**33** 付録・添付資料 32](#_Toc170767244)

# **研究の要約**

| 背景／研究計画の根拠 | 抗癌剤や分子標的薬投与時には高頻度で口腔粘膜炎が発症するが予防法は確立していない。今回、ベタメタゾン含嗽薬の口腔粘膜炎発症予防効果についてアズノールうがい薬を対照としてランダム化比較試験を実施する。 |
| --- | --- |
| 対象疾患 | 頭頸部がんと血液がんを除く固形癌で薬物療法を行う患者。 |
| 主要目的 | ベタメタゾン含嗽薬の口腔粘膜炎発症予防効果を検討する。 |
| 副次目的 | ベタメタゾン含嗽薬の口腔粘膜炎重症化予防効果を検討する。 |
| 主要評価項目 | ベタメタゾン含嗽薬のグレード1口腔粘膜炎発症予防効果についてアズノールうがい薬を対照として比較検討する。 |
| 副次評価項目 | ベタメタゾン含嗽薬のグレード２およびグレード3口腔粘膜炎発症予防効果についてアズノールうがい薬を対照として比較検討する。 |
| 研究デザイン | ランダム化比較試験 |

# **はじめに**

本研究はベタメタゾンを適応外使用するため、臨床研究法における特定臨床研究に該当する。

そのため、本研究に関わる者は臨床研究法を遵守して研究を実施する。

本臨床研究の実施について臨床研究審査委員会で審査し承認を得た上で実施医療機関の管理者による承認を得る。

【臨床研究審査委員会】

| 委員会名称 | 長崎大学臨床研究審査委員会  （The Clinical Research Review Board in Nagasaki University） |
| --- | --- |
| 設置者 | 国立大学法人長崎大学　学長 |
| 認定番号 | CRB7180001 |
| 住所 | 長崎県長崎市坂本１丁目７番１号  （1-7-1 Sakamoto, Nagasaki-shi, Nagasaki-ken） |
| 電話番号 | 095-819-7229 |
| メールアドレス | [gaibushikin@ml.nagasaki-u.ac.jp](mailto:gaibushikin@ml.nagasaki-u.ac.jp) |

# **研究の背景**

がん治療における薬物療法は、従来の殺細胞性抗がん剤に加え分子標的薬や免疫チェックポイント阻害薬の登場に伴い飛躍的に進歩してきた。薬物療法にはさまざまな有害事象があるが、重要な有害事象の一つに口腔粘膜炎がある。口腔粘膜炎は痛みを伴うことから経口摂食が不可能になり栄養状態に影響を与え、原疾患に対する治療の中断を余儀なくされることもあり、生命予後にまで影響を及ぼす可能性もある重大な有害事象である。しかしがん治療に伴う口腔粘膜炎に対する治療法や予防法は確立しておらず、MASCCのガイドライン[1]では、クライオセラピー、口腔ケア、鎮痛薬投与、亜鉛の投与などいくつかの対策が挙げられているが、エビデンスレベルの高い予防法、治療法はなく、食事形態の変更や局所麻酔薬による含嗽、オピオイド投与などの対症療法のみが行われているのが現状である。

頭頸部放射線治療時の口腔粘膜炎に対して1980年代にステロイド軟膏の有効性と安全性が報告された[2, 3]が、現在あまり普及していない。その理由の一つに、放射線治療時や薬物療法時にステロイドの局所投与を行うと口腔カンジダ症の発症を助長するのではないかという懸念があった[4]。これに対しわれわれは多施設共同観察研究において、頭頸部癌放射線治療時の口腔カンジダ症発症のリスク因子は白血球減少と粘膜炎の増悪であり、ステロイド軟膏は口腔カンジダ症発症を助長することはなく、むしろ有意差はないものの発症率を低下させることを示した[5]。しかし一方でステロイド軟膏は粘膜炎が咽頭粘膜まで広がった場合には塗布することが困難で、ステロイド含嗽薬のほうが使用しやすいが、本邦では粘膜炎に使用できるステロイド含嗽薬はない。

乳がん治療に用いられるエベロリムス投与時にはBOLERO-2 試験では 59％、日本人サブグループ解析では88.7%と高頻度で口腔粘膜炎を発症することが報告されている[6]。海外の試験でエベロリムス投与患者にデキサメタゾン含嗽を行ったところ、historical controlに比べてgrade 2以上の口腔粘膜炎の発症率は有意に減少することが報告された[7]。国内ではステロイド含嗽薬が使用できないため、研究分担者の梅田らはエベロリムス投与患者に対してデキサメタゾン軟膏塗布を含む歯科介入を行うと口腔粘膜炎の発症率が低下することを第Ⅲ相試験で明らかにした[8, 9]。また、久芳らは乳癌薬物療法時の口腔粘膜炎に対するステロイド含嗽は口腔粘膜炎の発症率を低下させることを明らかにした[10]。また殺細胞性抗癌剤投与時の口内炎に対するステロイド含嗽による局所投与は有効で安全であるとの報告もある[11]。

そこで今回われわれは薬物療法患者を対象としたランダム化第Ⅱ相試験によりステロイドの一つであるベタメタゾン含嗽の口腔粘膜炎予防効果を検討することとした。

# **研究の目的・意義**

「目的」

・本研究の目的はベタメタゾン含嗽により薬物療法に起因する口腔粘膜炎の発症や重症化を予防できるか検討することである。

「意義」

・本研究は薬物療法を受ける患者の口腔粘膜炎発症や重症化予防法を確立し患者のQOL向上に寄与することが期待されている。

# **研究の概要**

## 研究の方法

フェーズ（相）：2

研究の種類：介入研究

無作為化：ランダム化比較

盲検化：非盲検

対照：アズノール含嗽

割付け：並行群間比較

研究目的：予防

研究の位置づけ：探索的研究

## 被験薬／被験機器

本研究は下記の医薬品を被験薬・対照薬として用いる。

被験薬の管理については『4.3 被験薬の管理／被験機器の管理』に記載する。

【被験薬】

| 一般名 | ベタメタゾンリン酸エステルナトリウム |
| --- | --- |
| 商品名／製造販売業者 | リノロサール注射液20mg（0.4%）　（わかもと製薬） |
| 剤形・性状・含量 | 1アンプル5mL中ベタメタゾンリン酸エステルナトリウム26.3mg（ベタメタゾンとして20mg）  無色澄明の水性注射剤 |
| 薬効分類 | 合成副腎皮質ホルモン剤 |
| 効能・効果 | 抗炎症作用、抗アレルギー作用、免疫抑制作用 |
| 用法・用量 | 静脈内、点滴で静脈内、筋肉内、関節腔内、脊髄腔内、軟組織内、腱鞘内、鼻腔内などに注射または注入したり、ネブライザーで使用。ベタメタゾンとして、  〈静脈内注射〉  通常成人1回2～8mgを3～6時間毎に静脈内注射する。  〈点滴静脈内注射〉  通常成人1回2～10mgを1日1～2回注射する。  〈筋肉内注射〉  通常成人1回2～8mgを3～6時間毎に注射する。  〈関節腔内注射〉〈軟組織内注射〉〈腱鞘内注射〉〈滑液嚢内注入〉  〈硬膜外注射〉  通常成人1回2～10mgを注射する。原則として投与間隔を2週間以上とすること。  〈脊髄腔内注入〉〈胸腔内注入〉  通常成人1回1～5mgを週1～3回脊注入する。  〈卵管腔内注入〉  通常成人1回0.4～1mgを注入する。  〈注腸〉  通常成人1回0.4～6mgを注入する。  〈結膜下注射〉通常成人1回0.4～2mgを注射する。その際の液量は0.2～0.5mLとする。  〈球後注射〉  通常成人1回0.8～4mgを球後注射する。その際の液量は0.5～1.0mLとする。  〈点眼〉  通常成人1回0.25～1mg/mL溶液1～2滴を1日3～8回点眼する。  〈ネブライザー〉  通常成人1回0.1～2mgを1日1～3回ネブライザーで投与する。  〈鼻腔内注入〉〈副鼻腔内注入〉  通常成人1回0.1～2mgを1日1～3回注入する。  〈鼻甲介内注射〉  通常成人1回1～5mgを注射する。  〈鼻茸内注射〉  通常成人1回1～5mgを注射する。  〈喉頭・気管注入〉〈中耳腔内注入〉〈耳管内注入〉  ベタメタゾンとして、通常成人1回0.1～2mgを1日1～3回注入する。  〈食道注入〉通常成人1回1～2mgを注入する。  〈唾液腺管内注入〉  通常成人1回0.5～1mgを注入する。 |
| 保管条件 | 常温保存（1～30℃） |

【対照薬】

| 一般名 | アズレンスルホン酸ナトリウム水和物 |
| --- | --- |
| 商品名／製造販売業者 | アズノールうがい液4%（ロートニッテン株式会社）など＊ |
| 剤形・性状・含量 | 濃青色の液  1mL中アズレンスルホン酸ナトリウム水和物40mg含む |
| 薬効分類 | アズレン含嗽液 |
| 効能・効果 | 咽頭炎、扁桃炎、口内炎、急性歯肉炎、舌炎、口腔創傷 |
| 用法・用量 | アズレンスルホン酸ナトリウム水和物として、1回4〜6mg（1回押し切り分、又は5〜7滴）を、適量（約100mL）の水又は微温湯に溶解し、1日数回含嗽する。なお、年齢、症状により適宜増減する。 |
| 保管条件 | 常温保存（1～30℃） |

＊その他の後発医薬品（同一成分、同一剤形）も可とする。

## 被験薬／対照薬の管理

本研究で用いる被験薬は研究代表医師の科学研究費より購入し、各実施医療機関で適切に保管する。

処方する際は、研究代表医師、研究責任医師、あるいは研究分担医師から研究用として直接被験者に提供する。服用忘れによる未使用薬および期限切れ薬剤は、院内の薬剤廃棄手順に従って通常通り廃棄する。

　本研究で用いる対照薬は通常診療における処方箋にて処方する。

## 研究対象者の選定方針

以下の選択基準をすべて満たし、除外基準のいずれにも該当しない研究対象者を登録適格例とする。

【選択基準】

1. 研究参加施設において頭頸部がんと血液がんを除く固形癌で薬物療法を行う患者。抗がん剤、分子標的薬、免疫チェックポイント阻害薬の種類や組み合わせは問わない。
2. 同意取得時において、年齢が18歳以上の患者
3. 本研究への参加にあたり十分な説明を受けた後、十分な理解の上、患者本人の自由意思による文書同意が得られた患者。

●設定理由

1. 研究対象のため
2. 判断能力を有する患者を対象とするため
3. ヘルシンキ宣言の精神を遵守するため

【除外基準】

1. 過去にベタメタゾンリン酸エステルナトリウムに対して過敏症の既往がある患者
2. 含嗽不可の患者
3. すでに口腔粘膜炎が出現している患者
4. 口腔カンジダ症を発症している患者
5. その他、研究代表医師、研究責任医師または研究分担医師が研究対象者として不適当と判断した患者

●設定理由

1. 研究対象者の安全性を確保するため
2. 研究自体が行えないため
3. 本研究は口腔粘膜炎の予防効果をみるためすでに口腔粘膜炎が発症している患者は除外
4. ステロイド含嗽により口腔カンジダ症の重症化の可能性が懸念されるため
5. 研究対象者の安全性を確保する上で、また適正に研究を実施する上で、不適格な患者を研究医師等が除外できる余地を残すため。

## 治療（プロトコール治療）の概要

血液がんや頭頸部がんを除いた固形癌薬物療法患者を、2つのグループに分けて以下の検討を行う。

同意取得、登録後、ベタメタゾン含嗽群とアズノール含嗽群の2群にランダム割付を行い、4.10に示す項目を調査する。

登録

割付

ベタメタゾン含嗽

口腔粘膜炎なし

口腔カンジダ症発症なし

8週間経過観察

8週間経過観察

アズノール含嗽

・登録要件を満たした対象患者において、口腔粘膜炎や口腔カンジダ症を発症していないことを確認したら登録し、介入群と対照群の両者に1:1の割合でランダム化割り付けを行う。

・介入群は、通常の口腔管理^※^に加えて、ベタメタゾン含嗽10ml（0.01％）を、1日4回（毎食後^＊^、就寝前）、口に含んでブクブクを30秒～1分行う

・対照群は、通常の口腔管理^※^に加えて、アズノール含嗽10ml（4％）を1日4回（毎食後^＊^、就寝前）口に含んでブクブクを30秒～1分行う。

・両群とも口腔へのステロイド軟膏の使用は禁止とする。

・^＊^食事を取らない場合でも食事摂取相当時間に使用する

※通常の口腔管理：食事形態の変更、鎮痛薬あるいはオピオイド等の投与、口腔ケア、保湿剤の使用、など

※口腔カンジダ症判定方法

口腔内にカンジダ症を疑う症状がありがん治療担当医あるいは口腔管理担当医がカンジダ症と判断したもの。培養検査の有無や結果によらない。

## 治療（プロトコール治療）の基準

治療の途中で口腔カンジダ症の発症を認めた場合は、ベタメタゾン群では含嗽を休止する。カンジダ症が消退したら再開する。アズノール群では口腔カンジダ症を認めても含嗽の休止は行わない。

## 併用治療

本研究では併用が必須な薬剤、療法はない。

## 併用禁止薬／併用禁止機器／併用禁止療法

研究期間中は下記の薬剤の併用を禁止する。

口腔内へのステロイド軟膏

## 併用制限薬／併用制限機器／併用制限療法

本研究では併用制限薬、併用制限療法は設けない。

## 評価、収集項目とスケジュール

### スケジュール（表）

|  | 登録・割付^※1^ | 診察^※2^ | | | | 終了時 | 中止時 |
| --- | --- | --- | --- | --- | --- | --- | --- |
|  |  | 1週～2週に1回 | | | | 8週終了 |  |
| 同意取得 | ● | － | － | － | － | － | － |
| 割付 | ● | － | － | － | － | － | － |
| 情報収集1 | ● | － | － | － | － | － | － |
| 情報収集2 | － | ● | ● | ● | ● | － | － |
| 情報収集3 | － | － | － | － | － | ● | ● |

※1 登録日は、化学療法開始前でも開始後でも可

登録日と割付日は別日でも可

※2 入院加療では週1回または入院時、外来加療では加療日

クールの延期の場合は、延期に合わせて含嗽および情報収集を行う

歯科受診を必ずしも必須とせず、歯科医師が外来化学療法室などに訪れて診察することも許容する

### 観察・検査項目について

【登録時に収集する項目】

| 項目 | 詳細 |
| --- | --- |
| 患者背景 | 年齢、性別、既往歴と投与薬剤、原発部位、喫煙歴、飲酒歴、体重 |
| 治療関連因子 | 治療開始日、抗がん剤名、併用薬/併用療法 |
| 臨床検査所見  （院内検査） | 好中球数、リンパ球数、アルブミン、クレアチニン |
| 口腔所見 | 口腔粘膜炎発症の有無とgrade（CTCAE v3.0,およびv5.0）、残存歯数、義歯装着の有無、歯周検査値（歯周ポケット、出血、動揺）、歯垢付着度（OHI-S）、義歯清掃度（義歯装着の場合）、未処置歯の有無と部位、歯科X線を撮影している患者では歯槽骨吸収の程度 |

【割付後来院時に収集する情報】

| 項目 | 詳細 |
| --- | --- |
| 口腔粘膜炎 | 口腔粘膜炎発症の有無とgrade（CTCAE v3.0およびv5.0）、発症例では発症日 |
| カンジダ発症 | 発症の有無と時期 |
| 研究使用薬 | 使用状況  研究用薬に関連する有害事象（口腔内粘膜の発赤・腫脹などの炎症性変化）の有無 |
| 有害事象 | 味覚障害、口腔内感染症、その他の有害事象の発症の有無 |

【終了時・または中止時に収集する情報】

| 項目 | 詳細 |
| --- | --- |
| 治療関連因子 | 化学療法開始日～終了日、化学療法完遂の有無、鎮痛薬の使用、局所麻酔薬（含嗽・ゼリーなど）の使用、オピオイドの使用、好中球数（治療中最低値）、リンパ球数（治療中最低値）、アルブミン（治療中最低値）、クレアチニン（治療中最高値）、GCF-S製剤使用の有無と時期、PNI |
| 口腔粘膜炎 | 口腔粘膜炎発症の有無とgrade（CTCAE v3.0およびv5.0）、発症例では発症日 |
| カンジダ発症 | 発症の有無と発症日 |
| 研究使用薬 | 使用状況  研究用薬に関連する有害事象（口腔内粘膜の発赤・腫脹などの炎症性変化）の有無 |
| 有害事象 | 味覚障害、口腔内感染症、その他の有害事象の発症の有無 |

## 研究終了後の治療法

本研究で用いるベタメタゾン含嗽剤は、口腔粘膜炎に対して保険適応がないため、研究終了後に同じ治療を継続することができない。そのため、研究終了後は保険診療の範囲での別の治療の中から最善のものを選択し、起こりうる不利益が最小になるよう努める。

## 研究対象者への検査結果等の提供

患者のその後の治療に直接影響しないので研究対象者には希望がない限り通知しない。

## 個々の研究対象者における中止基準

【中止基準】

・研究対象者より中止の申し出があった場合

・有害事象の発生（原疾患の増悪、合併症の発生、新たな疾患の併発等）により、研究医師等が当該研究対象者に対する研究の継続を不適当と判断した場合

・その他、研究医師等が当該研究対象者に対して研究の継続が不適当と判断した場合

【中止後の対応】

　中止の場合は速やかにベタメタゾン含嗽剤の使用を中止し、後観察期間に移行して可能な限り最終来院までのフォローアップを行うように努める。中止後の治療については研究対象者毎に適切な治療を検討し、切り替える。

# **研究対象者に生じる予測される利益、不利益**

## 予測される利益

本研究へ参加することでベタメタゾンによる口腔粘膜炎の発症率を低下する可能性がある。また研究成果により将来の医療の進歩に貢献できる可能性がある。

## 予測される不利益

本研究に参加することによる直接の不利益は生じないが、ベタメタゾンに対するアレルギーとして発疹などの過敏症が発症する可能性がある。また口腔カンジダ症の発症や口腔感染症の増悪などの副作用が生じる可能性がある。これらの副作用が生じた場合は直ちに含嗽を中止し水でうがいをし、必要に応じて抗真菌薬の投与や抗菌薬の投与を検討する。

## 予測される副作用

ベタメタゾンに対する過敏症状

口腔カンジダ症の発症

口腔感染症の増悪

## 予測される不具合

該当なし

# **本研究に参加しない場合の治療法**

本研究で用いる被験薬は当該疾患に対して保険適応がないため、本研究に参加しない場合は保険適応内の治療法より選択し、患者へ提供する。

治療法としてはアズノールうがい液やデキサメタゾン軟膏などが挙げられる。

治療法を決定する際は提供する医療の利益、不利益を説明した上で、提供する。

| 治療方法 | 特徴・効能効果 | 代表的な副作用 |
| --- | --- | --- |
| アズノールうがい液 | 消炎作用 | 過敏症 |
| デキサメタゾン軟膏 | 口内炎の症状緩和 | 過敏症、口腔内感染症の悪化 |

# **目標症例数**

296名（ベタメタゾン含嗽群148例、アズノール含嗽群148例）

【設定根拠】

悪性腫瘍に対する薬物療法時の口腔粘膜炎発症率を40%と仮定し、久芳らの先行研究よりステロイド含嗽により口腔粘膜炎の発症リスクは0.68倍、27%になると仮定する。Fisherの正確確率検定で群間比較をおこない、両側αを0.2、powerを0.8とすると、必要症例数は266例（一群133例）になる。脱落を10%とし、目標投与症例数を296例（一群148例）に設定した。

# **研究実施期間**

研究実施期間：jRCT公開日～2026年12月31日

（症例登録期間：jRCT公開日～2026年3月31日）

（症例報告書作成締切日：2026年6月30日）

# **症例登録方法**

実施医療機関の研究責任医師または研究分担医師は、研究対象者から文書同意を得た後、「研究対象者スクリーニング名簿（対応表）」に必要事項を記入する。

実施医療機関の研究責任医師または研究分担医師は、対象患者が選択基準を全て満たし、除外基準のいずれにも該当しないことを確認し、症例登録票に必要事項を全て記入し、データマネジメント担当者：九州歯科大学　に症例登録票をメールする。

・症例登録先

・機関名：九州歯科大学

九州歯科大学で症例登録票を確認し、適格と判定された場合、登録結果として登録番号および割付結果が記載された症例登録確認書を発行する。症例登録確認書は、実施医療機関の研究責任医師または研究分担医師にメールにて送付される。

不適格と判定された場合、九州歯科大学は不適格と判定した理由とともに、実施医療機関の研究責任医師または研究分担医師に連絡する。

実施医療機関の研究責任医師または研究分担医師は九州歯科大学より受領した症例登録確認書を確認し、症例登録確認書で指示されたベタメタゾン含嗽またはアズノール含嗽を開始する。

# **無作為化の方法と割り付け調整因子**

割り付け方法：コンピュータソフトによりベタメタゾン含嗽群とアズノール含嗽群の2群に1：1の割合でランダムに割り付ける（層別ブロック法）。

割り付け因子：口内炎高リスク薬の有無（高リスク：添付文書に口内炎発症頻度が30%以上と記載されている薬剤）

登録・割付機関：九州歯科大学

# **盲検化の管理**

## 盲検化の方法

　該当なし

## 開鍵（キーオープン）の必要性の判断および手順

該当なし

# 評価項目

## 主要評価項目

Grade 1口腔粘膜炎発症の有無と発症日

## 副次評価項目

Grade 2および3口腔粘膜炎発症の有無と発症日

口腔カンジダ症発症および発症日

薬物療法完遂の有無

## 安全性評価項目

過敏症状の有無

口腔カンジダ症発症の有無

# **統計解析**

解析対象集団とその定義を以下に示す。解析対象集団はCONSORT声明に従い、フローチャートにして表現する。

| 解析対象集団 | 定義 |
| --- | --- |
| 登録例  Intention To Treat (ITT) | 本研究にランダム割付されたすべての研究対象者 |
| 安全性解析対象集団  Safety Analysis Set (SAS) | 研究用薬が使用されたすべての研究対象者 |
| 最大の解析対象集団  Full Analysis Set (FAS) | ITTのうち、研究用薬が使用され、主要評価項目が得られた研究対象者 |
| 研究計画書に適合した対象集団  Per Protocol Set (PPS) | FASのうち、研究計画書からの逸脱がない研究対象者。 |

## 解析対象集団の特定

【解析対象集団】ITT、FAS、PPS

患者背景について要約統計量を用いて要約する。

| 解析対象集団 | 定義 |
| --- | --- |
| 登録例  Intention To Treat (ITT) | 本研究にランダム割付されたすべての研究対象者 |
| 最大の解析対象集団  Full Analysis Set (FAS) | ITTのうち、研究用薬が使用され、主要評価項目が得られた研究対象者 |
| 研究計画書に適合した対象集団  Per Protocol Set (PPS) | FASのうち、研究計画書からの逸脱がない研究対象者。 |

## 主要評価項目の解析

【解析対象集団】FAS、PPS

ベタメタゾン含嗽群とアズノール含嗽群のgrade 1口腔粘膜炎の発症割合についてリスク差とリスク比およびそれらの95%信頼区間を推定する。事前のサンプルサイズ計算の設定に基づき、Fisherの正確確率検定より推定されたP値<0.2を統計的に有意と判断する。Kaplan-Meier法で時点ごとの累積発症率を推定し、参考のためにlog-rank testをおこなう。

## 副次評価項目の解析

【解析対象集団】FAS、PPS

1）それぞれの評価項目について要約する。

2）ベタメタゾン含嗽群とアズノール含嗽群のgrade 2口腔粘膜炎の発症割合についてリスク差とリスク比およびそれらの95%信頼区間を推定する。事前のサンプルサイズ計算の設定に基づき、Fisherの正確確率検定より推定されたP値<0.2を統計的に有意と判断する。Kaplan-Meier法で時点ごとの累積発症率を推定し、参考のためにlog-rank testをおこなう。

3）ベタメタゾン含嗽群とアズノール含嗽群の口腔カンジダ症発症率について、Kaplan-Meier法で算出しlog-rank testにより2群間の差の解析を行う。

4）各Gradeの口腔粘膜炎発生率に関連する因子について、Cox回帰分析によリ検討する。

5）多重性の調整は行わない。

## 安全性評価の解析

ベタメタゾン含嗽群とアズノール含嗽群の有害事象について記載する。

## 部分集団解析

該当なし

## 中間解析計画

実施しない

## 欠測データの取り扱い等

各症例において調査項目の一部に欠測値のあるデータも廃棄せず統計解析の対象とする。

## 統計解析計画の変更

本研究において、当初の統計解析計画からの変更がある場合は、研究計画書または統計解析計画書を改訂し、本研究の総括報告書において説明する。

# **データマネジメント**

## 症例報告書の種類

本研究では、症例報告書の作成に関して、紙媒体の症例報告書を採用する。

実施医療機関の研究責任医師または研究分担医師等は、対象者毎に症例報告書を作成する。症例報告書には計画書に求められているデータを記載し、「症例報告書の記入・修正の手引き」（作成する場合は添付・作成しない場合は計画書に記載）に従って、記載内容の変更または修正を行う。変更または修正を行う場合、実施医療機関の研究責任医師または研究分担医師等は、修正液等を使用せずに訂正箇所を二重線で消し、訂正箇所に捺印または署名し、訂正日を記入する。なお、訂正事項が「症例報告書の記入・修正の手引き」に規定する重要事項に該当する場合、訂正箇所に捺印または署名し、日付の他に訂正の理由も記入する。

症例報告書は、実施医療機関の研究責任医師が署名したものを原本として取り扱うものとし、実施医療機関の研究責任医師は記載されたデータが完全かつ正確であることを保証する。

観察期間中はデータを記載した症例報告書の写しを九州歯科大学に提出する。変更または修正があった場合は原本に上記の方法で訂正を行い、訂正を行った症例報告書の写しを再度九州歯科大学に提出する。

観察期間終了後、実施医療機関の研究責任医師は、すべての症例報告書の写しを作成したうえで、症例報告書の原本を郵送あるいは手渡しにて九州歯科大学に提出する。

写しは各実施医療機関において、本研究で規定する保管期間まで適切に保管する。

## 原資料の特定

本研究における原資料とは以下のものをいう。

・対象者の同意及び対象者への情報提供に関する記録

・診療録、画像フィルム等、症例報告書作成のもととなった記録

・その他研究実施医療機関で保管する本研究の実施に係る記録

## 症例報告書に直接記入され、かつ原資料と解すべき項目

本研究では下記の項目については症例報告書を原資料とする。

・臨床検査値に関し、基準値から外れているが臨床的に問題のある事象とは扱わない場合、そのように判断した旨と判断理由の記載

・有害事象に関し、追跡調査不要と判断した理由の記載

・併用薬、併用治療の使用理由の記載

# **疾病等の取り扱い**

## 用語の定義

【有害事象】

研究期間中に研究対象者に生じたあらゆる好ましくない、または意図しない徴候（臨床研究値の異常も含む）、症状または病気をいい、特定臨床研究の実施との因果関係の有無は問わない。

【疾病等】

特定臨床研究の実施に起因するものと疑われる疾病、障害、死亡又、感染症、臨床検査値の異常や諸症状を含む。

本研究では研究期間中に特定臨床研究の実施に起因するものと疑われる事象を疾病等として取り扱う。

## 疾病等の評価

発生した有害事象と研究との因果関係は、研究代表医師、研究責任医師又は分担医師が判断する。判断には介入治療開始との時間的関係だけでなく、基礎疾患の経過、合併症、併用薬、研究手順、事故及びその他の外的因子などに起因することも考慮して判断する。因果関係は以下の基準に従って判断し、記録する。

・因果関係有り又は否定できない – 当該臨床研究又は介入治療によって発現することが既知・未知に関わらず、以下に従って判断する。

➢研究又は介入治療に起因することが合理的である、または合理的な可能性がある

➢研究との間に時間的関係がある

➢他の原因が示せず、研究との因果関係が否定できない

・因果関係無し – 次の基準に従って判断する。

➢研究又は介入治療に起因することが合理的でない

➢時間的関係が示せない

➢その他の原因が示せる

## 予測できる疾病等

口腔カンジダ症発症

口腔内の感染症増悪

その他、被検薬の添付文書に記載されている副作用

## 疾病等が発現した場合の研究対象者への措置

実施医療機関の研究責任医師または研究分担医師は疾病等が発生した場合は、速やかに研究対象者へ適切な対応（説明等も含む）を行い、当該臨床研究の中止、中断、その他の必要な措置を講じる。

実施医療機関の研究責任医師または研究分担医師は最終観察時点で発生した疾病等が継続している場合はそれ以降も発生時前の状態に回復、あるいは臨床的に安定するまで経過を観察する。

## 疾病等の報告

実施医療機関の研究責任医師または研究分担医師は疾病等が発生した場合、事象名（診断名）、発現日、重症度、因果関係、予測性、転帰、転帰の判断日を記録する。

## 重篤な疾病等の報告

実施医療機関の研究責任医師は下記に該当する疾病等の発生を知った場合は速やかにそれぞれに定める提出期間内に実施医療機関の管理者、長崎大学臨床研究審査委員会に報告する。また、予測できない疾病等報告の際は、同時にそれぞれに定める期間内に厚生労働大臣（医薬品医療機器総合機構：PMDA）に報告する。


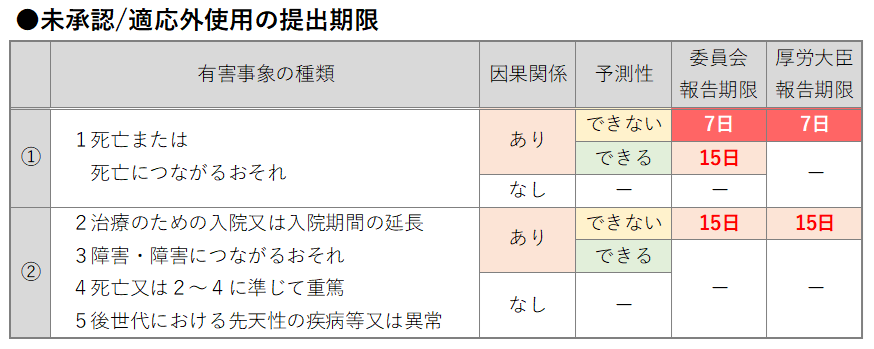


## 不具合の報告

該当なし

# **効果安全性評価委員会**

本研究では効果安全評価委員会は設置しない。

# **研究計画書の遵守、変更及び不適合（研究計画書からの逸脱等）**

## 研究計画書の遵守

実施医療機関の研究責任医師または研究分担医師は、研究代表医師の事前の合意及び実施計画に記載のある認定臨床研究審査委員会の審査に基づく文書による事前の承認を得ることなく、研究計画書に適合しないこと（すなわち研究計画書からの逸脱または変更）を行ってはならない。

## 研究計画書の変更

研究代表医師は研究計画書、その他、研究実施に係る事項について変更が生じた場合はその内容とその理由を認定臨床研究審査委員会に意見を聴いた上で、実施医療機関の管理者に承認を得る。

また、研究代表医師は実施計画（添付資料等も含む）に記載してある事項に変更が生じた場合はjRCTの情報を変更、更新し、厚生労働大臣に変更後の実施計画を提出する。

## 不適合の管理（研究計画書からの逸脱等）

1）実施医療機関の研究責任医師または研究分担医師は、研究で実施している事項が不適合であることを知った時は、速やかに実施医療機関の管理者に報告するとともに、これを研究代表医師に通知する。

2）研究代表医師は、研究対象者の人権、安全性及び研究の進捗並び結果の信頼性に影響を及ぼす重大なものが判明した場合においては、重大な不適合として、速やかに実施計画に記載のある認定臨床研究審査委員会の意見を聴く。

3）重大な不適合には、臨床研究の対象者の緊急の危険を回避するためその他、医療上やむを得ない理由により研究計画書に従わなかったものについては含まない。

# **インフォームド・コンセントについて**

## インフォームド・コンセントを受ける手続き

実施医療機関の研究責任医師または分担医師は、患者が研究に参加する前に、実施計画に記載のある認定臨床研究審査委員会で承認された同意・説明文書を用いて以下の事項を研究対象者へ十分に説明する。研究対象者が研究内容を十分理解したことを確認した後、参加の同意を文書により取得する。

説明書、署名された同意書の写しは速やかに研究対象者へ手渡す。

インフォームド・コンセントを受ける際に研究対象者等に対し説明すべき事項は、臨床研究法施行規則　第四十六条『特定臨床研究の対象者等に対する説明及び同意事項』に定められた事項とする。（同意説明文書参照）ただし、認定臨床研究審査委員会の意見を受けて研究機関の管理者が認めた事項については、この限りでない。

実施医療機関の研究責任医師または研究分担医師は、研究に継続して参加するかどうかについて対象者の意思に影響を与えると思われる情報を入手した場合には、直ちに当該情報を対象者に提供し、これを記録するとともに、対象者が研究に継続して参加するかどうかを確認する。また、研究責任医師が説明文書の改訂を行った場合は、研究責任医師または研究分担医師は、改訂された説明文書を用いて改めて説明し、研究への参加の継続について自由意思による同意を対象者から文書により得る。

また、研究実施医療機関の研究責任医師が必要と判断した場合、施設ごとに説明文書・同意書を変更することができるが、その場合は研究代表者に提出を行い、実施計画に記載のある認定臨床研究審査委員会の承認を得る。

## 研究対象者等及びその関係者からの相談等への対応

研究対象者等やその関係者からの相談には、研究実施機関の研究責任医師または研究分担医師または研究事務局が対応する。

## 代諾者等からインフォームド・コンセントを受ける場合

該当なし

## インフォームド・アセント

該当なし

## 研究対象者等に対する同意取得が不要な場合

　該当なし

# **個人情報等の取り扱い**

## 個人情報の管理について

本研究では研究対象者を登録する際に、研究対象者識別コードを付与する。研究対象者識別コードは、イニシャルやカルテID等のような特定の個人を識別できる情報とは無関係の数字記号等で構成され、症例登録票や症例報告書等の本研究に関する書類を作成する際には研究対象者識別コードを使用することで匿名化を行う。研究責任医師は、匿名化された情報から、必要に応じて研究対象者を識別することができるように研究対象者の氏名やカルテID等の情報が記載された対応表を作成し、外部に漏洩することがないよう厳重に保管、管理を行う。本研究に関わる関係者は、対象者の個人情報保護について、適用される法令、条例を遵守する。

また関係者は、対象者の個人情報およびプライバシー保護に最大限の努力を払い、本研究を行う上で知り得た個人情報を正当な理由なく漏らしてはならない。関係者がその職を退いた後も同様とする。

本研究は多施設共同研究であり、各医療機関で対応表を適切に管理することとし、実施医療機関外への提供は行わない。

研究対象者の症例登録票、症例報告書、検査データ等を研究機関外に提供する際には、研究対象者識別コードまたは登録番号を用い、匿名化する（どの研究対象者の試料・情報であるかが直ちに判別できないよう、加工または管理されたものに限る）。本研究における個人情報等は、本研究計画書のほか、各実施医療機関における臨床研究に関する個人情報等の取扱いに関する手順書を遵守して取り扱う。

## 保有個人情報のうち本人を識別することができるものの開示

研究責任医師は、本人等から、保有個人情報のうち本人を識別することができるものについて開示を求められた場合には、その求めをした本人等に対し、遅滞なく、該当する個人情報を開示しなければならない。ただし、開示することにより次のいずれかに該当する場合は、その全部又は一部を開示しない場合がある。

一　本人又は第三者の生命、身体、財産その他の権利利益を害するおそれがある場合

二　臨床研究の適正な実施に著しい支障を及ぼすおそれがある場合

三　他の法令に違反することとなる場合

## 遺伝的特徴等に関する情報の取扱い（偶発的所見を含む）

本研究では研究対象者の遺伝的特徴が得られるような検査、解析は実施しないため、該当しない。

## 原資料等の閲覧

本研究では、研究代表医師および実施医療機関が、当該臨床研究に関連するモニタリング、監査並びに認定臨床研究審査委員会及び規制当局の調査の際に、原資料等の全ての臨床研究関連記録を直接閲覧に供する。

# **情報の保管および破棄の方法**

本研究では生体試料の保管は行わない。研究代表医師は、下記に掲げる本研究に関する文書および記録を施錠可能な保管庫で厳重に保管管理する。

電子データで保管する場合は、パスワードを設定した上で、院内LANやインターネットから独立したパソコンまたはUSBメモリ等の電磁的記録媒体にて保管し、使用していない時は施錠可能な保管庫で厳重に保管管理する。保管期間は、本研究が終了した日から５年間とする。保管期間を経過した文書および記録は、個人情報や機密情報の漏洩がないように細心の注意を払い廃棄する。紙媒体はシュレッダーにて裁断し廃棄する。

その他の媒体に関しては、匿名化の上、削除等の適切な方法により廃棄する。

実施医療機関の研究責任医師は実施医療機関の規程に従い、下記の文書を研究終了後５年間保管する。

1）研究対象者を特定する事項（対応表）＊

2）研究対象者に対する診療及び検査に関する事項

3）特定臨床研究への参加に関する事項（症例登録票）（原本）

4）研究計画書

5）実施計画

6）説明文書・同意書・同意撤回書

7）同意書（署名ありの原本）＊

8）症例報告書（原本）

9）実施医療機関の管理者の承認を得るために提出した書類および実施承認書

10）モニタリング及び監査（監査を実施する場合）に関する文書

11）特定臨床研究の実施に係る契約書（写）

（医薬品等製造販売業者またはその特殊関係者と締結した契約に係るものを除く。）

13）その他本研究に関連する文書または記録

＊実施医療機関ごとに保管し、他機関への提供は行わないこと

# **情報の二次利用**

本研究の目的以外に、本研究で得られた試料および情報等を利用する予定はないが、本研究で得られたデータをUMIN-ICDR（https://www.umin.ac.jp/icdr/index-j.html）に寄託する可能性がある。

# **情報のバイオバンクとしての利用**

該当なし

# **研究の資金源等、研究に係る利益相反管理**

## 研究の資金源等

本研究は、科学研究費（基盤C、令和5～7年度）の研究助成を得て実施する（助成番号23K09315）。

## 利益相反管理

研究代表医師は、本研究の利益相反管理基準を作成する。研究代表医師は、利益相反申告者の臨床研究への企業の関与の内容を確定させ、関係企業等報告書を作成する。

利益相反申告者は、実施医療機関の管理者に事実関係の確認を行い、利益相反確認報告書を入手する。研究代表医師は、利益相反確認報告書の内容を踏まえ、利益相反管理計画を作成し、実施計画に記載のある認定臨床研究審査委員会の意見を聴き、適切な管理を行う。

# **研究対象者の費用負担・謝礼について**

本研究の実施にかかる費用のうち、個々の患者に要する医療費（診察費、入院費用、薬剤費、検査代など）については、本研究で行う治療がすべて保険診療範囲であることから自己負担分を研究対象者が支払う。本研究へ参加することで研究対象者に別途金銭的負担が増えることはない。本研究では、研究対象者に対して金銭的およびそれ以外による参加謝礼並びに負担軽減費等の支払い等は行わない。

# **健康被害に対する補償**

本研究は重篤な有害事象の発生は非常にまれと考えられるため、臨床研究賠償責任保険への加入は行わない。

本研究の実施に起因して健康被害が発生した場合には、研究責任医師、または研究分担医師は、研究対象者に対し適切な治療・処置をおこなう。

治療は原則として通常の保険診療と同様に、研究対象者の健康保険を用いておこなう。その際の医療費の自己負担分の支払い、休業補償、差額ベッド料金などの金銭的な補填は行わない。

# **定期報告**

## 認定臨床研究審査委員会／管理者への定期報告

研究代表医師は、特定臨床研究の実施状況について、実施計画を厚生労働大臣に提出した日から起算して１年ごとに（当該期間満了後２月以内）、自機関の管理者に報告した上で、当該実施計画に記載された認定臨床研究審査委員会に定期報告を行う。報告事項は以下のものとする。

(1) 参加した臨床研究対象者の数

(2) 疾病等の発生状況及びその後の経過

(3) 不適合の発生状況及びその後の対応

(4) 安全性及び科学的妥当性についての評価

(5) 利益相反管理基準に定める医薬品等製造販売業者等の関与に関する事項

また、研究代表医師は認定臨床研究審査委員会への報告後、速やかに実施医療機関の管理者に報告しなければならない。

研究代表医師は、認定臨床研究審査委員会に報告を行ったときは、その旨を、速やかに他の研究責任医師に情報提供を行う。当該他の研究責任医師は、速やかに当該情報提供の内容を各実施医療機関の管理者に報告する。

## 厚生労働大臣への定期報告

研究代表医師は、特定臨床研究の実施状況について、実施計画に記載された認定臨床研究審査委員会が意見を述べた日から起算して１ヶ月以内に、以下の事項について厚生労働大臣に報告する。

(1) 実施計画に記載されている委員会の名称

(2) 委員会による当該特定臨床研究の継続の適否

(3) 特定臨床研究に参加した特定臨床研究対象者の数

# **研究の終了と中止**

## 研究の終了

『28.2 研究結果の公表』参照

## 研究の中止

【中止基準】

下記のような状況が発生した場合、研究を中止する場合がある。

・予測できない重篤な疾病等が発生し、研究対象者全体への不利益が懸念される場合

・法及び関連法令または研究計画書に対する重大な違反／不遵守が判明した場合

・倫理的妥当性もしくは科学的合理性を損なう、または損なう恐れのある事実を得た場合

・研究対象者に対する重大なリスクが特定された場合

・認定臨床研究審査委員会より意見を述べられた場合

・厚生労働大臣より中止要請や勧告を受けた場合

【中止の手続き】

研究代表医師は研究を中止する場合、中止の日から10日以内に、その旨を研究代表医師は認定臨床研究審査委員会へ通知するとともに厚生労働大臣に中止届書を届け出る。

また必要に応じて中止について認定臨床研究審査委員会の意見を聴く。

中止届書を厚生労働大臣に提出後も臨床研究が終了するまでは定期報告を継続的に提出し、当該臨床研究の進捗状況に関する事項の変更に該当する場合には、実施計画の変更の届出を行う。

【研究対象者への対応】

研究責任医師等は研究対象者と連絡をとり、研究スケジュールの中止について伝え、今後の対応、来院スケジュールについて説明する。中止する場合は研究対象者への倫理的配慮、安全性を十分に検討し、リスクを最小限に収めるよう努める。

# **研究の情報公開及び結果の公表**

## 研究の登録

本研究の実施に先立ち、厚生労働省が整備するデータベース（jRCT = Japan Registry of Clinical Trials）に記録（登録）する。

研究計画書の変更及び研究の進捗に応じて適宜更新し、研究を終了したときは、研究の結果を登録する。

## 研究結果の公表

研究代表医師は、全て評価項目に係るデータの収集を行うための期間が終了した１年以内には総括報告書及びその概要を作成する。

（中止した日または全ての評価項目に係るデータの収集を行うための期間が終了した日のいずれか遅い日から原則一年以内に総括報告書を作成、提出。）

総括報告書及びその概要を作成した時は認定臨床研究審査委員会の意見を聴き、当該委員会が意見を述べた日から起算して１月以内に実施医療機関の管理者に提出するとともに総括報告書の概要を厚生労働大臣に提出しjRCTで公表する。

総括報告書の概要がjRCTに公開された日が研究終了日となる。

jRCTに公開されたら研究代表医師はその旨を速やかに実施医療機関の管理者に報告する。

研究代表医師は、実施医療機関の管理者への提出時に、その旨を他の研究責任医師に情報提供し、当該他の研究責任医師は、速やかに、当該情報提供の内容を他の実施医療機関の管理者に報告する。また、総括報告書の概要がjRCTに公表された場合も速やかに情報提供を行う。

## 28.3特定臨床研究の個々の対象者の匿名化されたデータを共有

他の研究者より合理的な理由に基づいて共有の依頼があった場合はその研究者に提供する可能性もある。

# **品質管理及び品質保証**

## モニタリング

研究代表医師は本研究が研究対象者の人権、安全が確保されている事、実施計画、研究計画書及び省令を遵守して実施されていること、データの信頼性等が確保されている事を確認するために、指名したモニタリング担当者にモニタリングを行わせる。

モニタリングの実施等に係る事項については別途作成する「モニタリング手順書（計画書）」に規定する。モニタリング担当者は「モニタリング手順書（計画書）」に従いモニタリングを実施する。

## 監査

本研究では、監査を実施しない。

# **研究成果の帰属（知的財産権）**

本研究から特許権、またそれを基として経済的利益が生じる可能性があるが、その権利は研究を実施する研究機関や研究責任医師等に帰属し、研究対象者がこの権利を持つことはない。

また本研究計画書に基づいて行われた研究成果は、長崎大学のものとする。

# **研究の実施体制**

## 研究代表医師

所属：長崎大学病院

## 研究事務局

設置しない

## 実施医療機関および研究責任医師

実施計画（様式第一）1（4）」参照

## データマネジメント責任者

所属：九州歯科大学

## 統計解析責任者

なし

## モニタリングに関する責任者

所属：長崎大学病院

## 監査に関する責任者

なし

## 研究・開発計画支援担当者

なし

## 調整管理事務担当者

なし

## 研究責任医師または研究代表医師以外に研究を総括する者

所属：愛知学院大学

## その他臨床研究に関連する臨床検査施設並びに医学的及び技術的部門・機関

なし

## 開発業務受託機関

なし

# 文献

1. Lalla RV, et al. MASCC/ISOO clinical practice guidelines for the management of mucositis secondary to cancer therapyCancer, 120: 1453-1461, 2014.

2. 上野　正, 他. 二重盲検法によるデキサルチン軟膏の臨床試験成績．日口外誌, 26:1399-1408,1980.

3. 手島昭樹, 他. 頭頸部腫瘍患者の放射線口内炎に対するケナログ軟膏の有効性、安全性についての検討．薬理と治療, 14:7163-7166,1986.

4. 全国共通がん医科歯科連携講習会テキスト第2版.2018.

5. Nshii M, et al. Factors associated with severe oral mucositis and candidiasis in patients undergoing radiotherapy for oral and oropharyngeal carcinomas: a retrospective multicenter study of 326 patients. Support Care Cancer, 28(3): 1069-1075, 2020.

6. Baselga J, et al. Everolimus in postmenopausal hormone-receptor-positive advanced breast cancer. N Engl J Med,366: 520-529, 2012.

7. Rugo HS, et al. Prevention of everolimus-related stomatitis in women with hormone receptor-positive, HER2-negative metastatic breast cancer using dexamethasone mouthwash (SWISH): a single-arm, phase 2 trialLancet Oncol, 18: 654-662, 2017.

8. Niikura, et al. Oral Care Evaluation to Prevent Oral Mucositis in Estrogen Receptor-Positive MetastaticBreast Cancer Patients Treated with Everolimus (Oral Care-BC): A Randomized Controlled Phase III Trial. Oncologist, 25(2):223-e230 2020.

9. Umeda, et al. Oral care and oral assessment guide in breast cancer patients receiving everolimus and exemestane: subanalysis of a randomized controlled trial (Oral Care-BC). Ann Transl Med, 9(7): 535 2021.

10. Kuba S, et al. Efficacy and safety of a dexamethasone-based mouthwash to prevent chemotherapy-induced stomatitis in women with breast cancer: A multicenter, open-label, randomized phase 2 study. J Evid-Based Dent Pract, 23:101896. 2023.

11. Fernández-Sala X、et al. Effectiveness and safety of a novel dexamethasone mouthwash formulation in managing stomatitis in cancer patients. Farm Hosp.　45:41-44, 2020．
