# Supplementary material for: Prevention of oral mucositis in patients undergoing cancer chemotherapy using betamethasone mouthwash: A multicenter randomized controlled trial protocol
Source: PLoS One. 2026 Apr 8;21(4):e0345991. doi: 10.1371/journal.pone.0345991 (PMC13061232; doi:10.1371/journal.pone.0345991)
Supplement: S3 File — (DOCX) [file pone.0345991.s003.docx]

A multicenter randomized controlled trial of betamethasone-based mouth wash for the prevention of oral mucositis

**Principal Investigator:**

**Nagasaki University Hospital**

**Creation Date: June 18, 2025**

**Version Number: 2.1**

table of contents

1. [Summary of the study 6](#_bookmark0)
2. [At first 7](#_bookmark1)
3. [Research Background 7](#_bookmark2)
4. [Purpose and Significance of Research 8](#_bookmark3)
5. [Outline of Research 8](#_bookmark4)
   1. [Methods of research 8](#_bookmark5)
   2. [Test Drug/Device 9](#_bookmark6)
   3. [Subject/control drug management 11](#_bookmark7)
   4. [Selection Policy for Research Subjects 11](#_bookmark8)
   5. [Overview of treatment (protocol treatment) 12](#_bookmark9)
   6. [Criteria for treatment (protocol treatment) 13](#_bookmark10)
   7. [Concomitant treatment 13](#_bookmark11)
   8. [Prohibited Drugs / Devices / Therapies 13](#_bookmark12)
   9. [Combined use of limiting shalf/combined use of limiting machine/combined use of limiting](#_bookmark13) [therapy13](#_bookmark13)
   10. [Assessments, collection items and schedules 13](#_bookmark14)
       - [Schedule (Table) 13](#_bookmark15)
       - [Observation and Inspection Items 14](#_bookmark16)
   11. [Treatment after the end of the study 15](#_bookmark17)
   12. [Provision of test results, etc. to research subjects 15](#_bookmark18)
   13. [Discontinuation criteria in individual study subjects 16](#_bookmark19)
6. [Predicted benefits and disadvantages for the study subject 16](#_bookmark20)
   1. [Projected Profits 16](#_bookmark21)
   2. [Foreseeable disadvantages 16](#_bookmark22)
   3. [Expected side effects 16](#_bookmark23)
   4. [Expected side effects 16](#_bookmark24)
7. [Treatment if not participating in the present study 17](#_bookmark25)
8. [Target number of cases 17](#_bookmark26)
9. [Duration of the study 17](#_bookmark27)
10. [How to Register a Case 17](#_bookmark28)
11. [Randomization Method and Allocation Adjustment Factors 18](#_bookmark29)
12. [Managing Blinding 18](#_bookmark30)
    1. [Methods of blinding 18](#_bookmark31)
    2. [Determination and procedure for the need for key opening (key opening) 18](#_bookmark32)
13. [Evaluation items 19](#_bookmark33)
    1. [Primary endpoint 19](#_bookmark34)
    2. [Secondary endpoints 19](#_bookmark35)
    3. [Safety endpoints 19](#_bookmark36)
14. [Statistical analysis 19](#_bookmark37)
    1. [Identify the target population 19](#_bookmark38)
    2. [Primary endpoint analysis 20](#_bookmark39)
    3. [Analysis of secondary endpoints 20](#_bookmark40)
    4. [Analysis of Safety Assessment 20](#_bookmark41)
    5. [Subpopulation analysis 20](#_bookmark42)
    6. [Interim Analysis Plan 20](#_bookmark43)
    7. [Handling of missing data, etc 21](#_bookmark44)
    8. [Changing the Statistical Analysis Plan 21](#_bookmark45)
15. [Data management 21](#_bookmark46)
    1. [Types of case report forms 21](#_bookmark47)
    2. [Identification of source materials 21](#_bookmark48)
    3. [Items that are entered directly in the case report form and should be interpreted as source](#_bookmark49) [materials 22](#_bookmark49)
16. [Handling of diseases, etc 22](#_bookmark50)
    1. [Definition of terms 22](#_bookmark51)
    2. [Evaluation of diseases, etc. 22](#_bookmark52)
    3. [Predictable diseases, etc. 23](#_bookmark53)
    4. [Measures to be taken for research subjects in the event of illness, etc. 23](#_bookmark54)
    5. [Reporting of illnesses, etc 23](#_bookmark55)
    6. [Reporting of serious illnesses, etc 23](#_bookmark56)
    7. [Reporting Defects 23](#_bookmark57)
17. [Effectiveness and safety evaluation committee 24](#_bookmark58)
18. [Compliance, modification and non-conformity with the research plan (deviation from the](#_bookmark59) [research plan, etc.) 24](#_bookmark59)
    1. [Compliance with the research protocol 24](#_bookmark60)
    2. [Changes to the research proposal 24](#_bookmark61)
    3. [Management of non-compliance (Deviations from the study protocol) 24](#_bookmark62)
19. [About informed consent 24](#_bookmark63)
    1. [Procedure for obtaining informed consent 24](#_bookmark64)
    2. [Handling of inquiries from participants or their relatives 25](#_bookmark65)
    3. [When obtaining informed consent from a substitute, etc 25](#_bookmark66)
    4. [Informed ascent 25](#_bookmark67)
    5. [Cases where informed consent is not required 25](#_bookmark68)
20. [Handling of personali information, etc 25](#_bookmark69)
    1. [Management of Personal Information 25](#_bookmark70)
    2. [Disclosure of retained personal information that can be used to identify the individual 26](#_bookmark71)
    3. [Handling of information on genetic characteristics, etc. (including accidental findings) 26](#_bookmark72)
    4. [Access to source documents 26](#_bookmark73)
21. [Storage and disposal of information 27](#_bookmark74)
22. [Secondary use of information 27](#_bookmark75)
23. [Use as a biobank of information 27](#_bookmark76)
24. [Sources of funding and conflict of interest management 28](#_bookmark77)
    1. [Sources of funding for the research, etc 28](#_bookmark78)
    2. [Managing Conflicts of Interest 28](#_bookmark79)
25. [Participant Costs and Compensation 28](#_bookmark80)
26. [Compensation for health hazards 28](#_bookmark81)
27. [Periodic Reporting 28](#_bookmark82)
    1. [Periodic reporting to accredited clinical research review boards/administrators 28](#_bookmark83)
    2. [Periodic Report to the Minister of Health, Labour and Welfare 29](#_bookmark84)
28. [Termination and Discontinuation of the Study 29](#_bookmark85)
    1. [Termination of study 29](#_bookmark86)
    2. [Discontinuation of the study 29](#_bookmark87)
29. [Disclosure of research information and results 30](#_bookmark88)
    1. [Enrollment of studies 30](#_bookmark89)
    2. [Publication of research results 30](#_bookmark90)
    3. [Sharing anonymized data of individual subjects of specific clinical studies 31](#_bookmark91)
30. [Quality Control & Quality Assurance 31](#_bookmark92)
    1. [monitoring 31](#_bookmark93)
    2. [audit 31](#_bookmark94)
31. [Attribution of Research Results (Intellectual Property Rights) 31](#_bookmark95)
32. [Research Implementation Structure 31](#_bookmark96)
    1. [Principal investigator 31](#_bookmark97)
    2. [Research Secretariat 32](#_bookmark98)
    3. [Participating Medical Institutions and Responsible Investigator 32](#_bookmark99)
    4. [Data Management Officer 32](#_bookmark100)
    5. [Statistical Analysis Officer 32](#_bookmark101)
    6. [Monitoring Officer 32](#_bookmark102)
    7. [Audit Officer 32](#_bookmark103)
    8. [R&D Planning Support Officer 32](#_bookmark104)
    9. [Coordination and administrative staff 32](#_bookmark105)
    10. [A person who supervises the research other than the principal investigator or the principal](#_bookmark106) [investigator 33](#_bookmark106)
    11. [Other clinical laboratories related to clinical research, as well as medical and technical](#_bookmark107) [departments and institutions 33](#_bookmark107)
    12. [Contract Development Organizations 33](#_bookmark108)
33. [References 33](#_bookmark109)
34. [Appendices and Attachments 34](#_bookmark110)

# Summary of the study

| Background/Rationale for the Research Plan | Oral mucositis occurs frequently when anticancer drugs and molecularly targeted drugs are administered, but prevention methods have not been established. In this study, we conducted a randomized controlled trial of the effect of betamethasone-containing drugs on preventing the onset of oral mucositis, using Aznol mouthwash as a control. |
| --- | --- |
| Target Diseases | Patients receiving drug therapy with solid tumors except head and neck cancers and blood cancers. |
| Primary Objective | The effect of betamethasone-containing drugs on preventing the development of oral mucositis will be examined. |
| Secondary Objectives | The effect of betamethasone-containing drugs on preventing the aggravation of oral mucositis will be examined. |
| Primary endpoint | The effect of betamethasone-containing drugs on preventing the onset of grade 1 oral mucositis will be compared with Aznol mouthwash as a control. |
| Secondary endpoints | The efficacy of betamethasone-containing drugs in preventing the development of grade 2 and grade 3 oral mucositis was compared with aznol mouthwash as a control. |
| Study design | Randomized controlled trials |

# At first

This study involves the off-label use of betamethasone and is categorized as a specified clinical trial under the Clinical Trials Act.

Thus, all parties involved in the study must adhere to the Clinical Trials Act during its implementation. The study will proceed after receiving approval from the Clinical Research Review Board and authorization from the administrators of the participating medical institutions.

[Clinical Research Review Committee]

| Name of the Committee | Nagasaki University Clinical Research Review Board  （The Clinical Research Review Board in Nagasaki University） |
| --- | --- |
| Installer | President, Nagasaki University |
| Certification Number | CRB7180001 |
| address | Nagasaki, Nagasaki, Sakamoto 1-7-1  (1-7-1 Sakamoto, Nagasaki-shi, Nagasaki-ken) |
| telephone number | 095-819-7229 |
| E-mail address | [gaibushikin@ml.nagasaki-u.ac.jp](mailto:gaibushikin@ml.nagasaki-u.ac.jp) |

# Research Background

Pharmacotherapy in cancer treatment has advanced dramatically with the introduction of molecular targeted therapies and immune checkpoint inhibitors, in addition to conventional cytotoxic anticancer agents. While pharmacotherapy has various adverse events, one significant adverse event is oral mucositis. Oral mucositis can cause severe pain, leading to the inability to consume food orally, which affects the nutritional status and may force the interruption of treatment for the primary disease, potentially affecting survival outcomes. However, there are no established treatments or preventive measures for oral mucositis associated with cancer therapy. The MASCC guidelines [1] suggest several measures, such as cryotherapy, oral care, analgesics, and zinc supplementation. However, there are no high-evidence preventive or therapeutic methods, and the current standard consists of symptomatic treatments like dietary modifications, local anesthetics, and opioid administrationThe efficacy and safety of steroid ointments for oral mucositis during head and neck radiation therapy were reported in the 1980s [2, 3], but it has not been widely adopted. One reason for this is the concern that local steroid application during radiation therapy or pharmacotherapy may promote the onset of oral candidiasis [4]. In response, we conducted a multicenter observational study showing that the risk factors for oral candidiasis during head and neck cancer radiation therapy were leukopenia and worsening mucositis. Our findings indicated that steroid ointment did not promote oral candidiasis but actually reduced its incidence, although the difference was not statistically significant [5]. However, steroid ointments are difficult to apply when mucositis extends to the pharyngeal mucosa, making steroid gargles easier to use. Unfortunately, no steroid gargles for mucositis are available in Japan.

In the BOLERO-2 trial, the incidence of oral mucositis during everolimus therapy for breast cancer was reported as 59%, with a higher incidence of 88.7% in a Japanese subgroup analysis [6]. A foreign trial demonstrated that dexamethasone gargles significantly reduced the incidence of Grade 2 or higher oral mucositis in everolimus-treated patients compared to a historical control group [7]. In Japan, where steroid gargles are not available, Umeda et al. showed in a phase III trial that the incidence of oral mucositis in patients treated with everolimus was reduced through dental interventions, including the application of dexamethasone ointment [8, 9]. Additionally, Hisahashi et al. showed that steroid gargles reduced the incidence of oral mucositis during pharmacotherapy for breast cancer [10]. There are also reports that local steroid gargle administration is effective and safe for treating oral mucositis induced by cytotoxic anticancer agents [11]. Given this background, we decided to evaluate the preventive effect of betamethasone gargle, a type of steroid, on oral mucositis in patients undergoing pharmacotherapy, through a randomized phase II trial.

# Purpose and Significance of Research

#### Purpose

The purpose of this study is to examine whether betamethasone gargles can prevent the onset or worsening of oral mucositis caused by pharmacotherapy.

#### Significance

This study aims to establish a preventive method for oral mucositis in patients undergoing pharmacotherapy, thereby contributing to improved quality of life (QOL) for these patients.

# Outline of Research

- 1. Methods of research

**Phase:** II

**Study Type:** Interventional Study

**Randomization:** Randomized Controlled Trial

**Blinding:** Open-label

**Control:** Azulene Gargle

**Allocation:** Parallel Group Comparison

**Study Objective:** Prevention

**Study Positioning:** Exploratory Study

- 1. Test Drug/Device

In this study, the following drugs were used as test drugs and control drugs.

The management of the test drug is described in "4.3 Management of the test drug / Management of the test device".

[Test drug]

| Generic name | Betamethasone sodium phosphate |
| --- | --- |
| Product Name /  Manufacturer / Distributor | Linolosal Injection 20mg (0.4%) (Wakamoto Pharmaceutical) |
| Dosage form, properties, and content | Sodium betamethasone phosphate in 1 ampoule 5 mL 26.3 mg (20 mg as betamethasone)  Clear, colorless aqueous injection |
| Medicinal classification | Synthetic corticosteroids |
| Indications | Anti-inflammatory effect, anti-allergy effect, immunosuppressive effect |
| Dosage | Injected or injected intravenously, intramuscularly, intraarticularly, intraspinal cavity, soft tissue, tendon sheath, nasal cavity, etc., or used in a nebulizer. As betamethasone,  〈Intravenous injection〉  Adults are usually injected intravenously at 2~8mg once every 3~6 hours.  〈Intravenous injection〉  Adults are usually injected 2~10mg 1~2 times a day.  〈Intramuscular injection〉  Normally, 2~8mg is injected every 3~6 hours for adults.  〈Intraluminal injection〉〈Intra-soft tissue injection〉〈Intra-tendon injection〉〈Intrasynovial fluid injection〉  〈Epidural injection〉  Adults are usually injected 2~10mg at a time. As a general rule, the interval between doses should be at least 2 weeks.  〈Intra-spinal injection〉〈Intrathoracic injection〉  Normally, 1~5mg per adult is injected 1~3 times a week.  〈Intralumen injection〉  Usually 0.4~1mg is injected once for adults.  〈Enema〉  Usually 0.4~6mg is injected once an adult.  〈Subconjunctival injection〉 Usually 0.4~2mg per adult injection. The amount of liquid at that time is 0.2 ~ 0.5 mL.  〈Postbulbar injection〉  Normally, 0.8~4mg is injected once for adults. The amount of liquid at that time shall be 0.5 ~ 1.0 mL.  〈Eye drops〉  Normally, 1~2 drops of 0.25~1mg/mL solution are instilled 3~8 times a day for adults.  〈Nebulizer〉  Usually, 0.1~2mg per adult is administered with a nebulizer 1~3 times a day.  〈Intranasal injection〉〈Paranasal injection〉  Adults usually inject 0.1~2mg 1~3 times a day.  〈Intranasal turbinate injection〉 Normally, 1~5mg is injected once per adult.  〈Nasal mushroom injection〉  Normally, 1~5mg is injected once per adult.  〈Laryngeal and tracheal injection〉〈Intra-middle ear cavity injection〉〈Eustachian tube injection〉  As betamethasone, it is usually injected 0.1~2mg once a day for adults 1~3 times a day.  〈Esophageal injection〉 Normally, 1~2mg is injected once per adult.  〈Injection into the salivary gland duct〉 Usually 0.5~1mg is injected once for adults. |
| Storage conditions | Store at room temperature (1~30°C) |

[Control drug]

| Generic name | Sodium azulene sulfonate hydrate |
| --- | --- |
| Product Name / Manufacturer /  Distributor | Aznol mouthwash 4% (Rothnitten Co., Ltd.), etc.* |
| Dosage form, properties, and  content | Dark blue liquid  Contains 40 mg of sodium azulene sulfonate hydrate in 1 mL |
| Medicinal  classification | Azulene-containing solution |
| Indications | Pharyngitis, tonsillitis, endostomatitis, acute flesh inflammation, glossitis, oral wounds |
| Dosage | As sodium azulene sulfonate hydrate, 4 to 6 mg (one press cut or 5 to 7 drops) is dissolved in an appropriate amount (about 100 mL) of water or slightly warm water, and soaking several times a day. In addition, it may be increased or decreased as appropriate depending on age and symptoms. |
| Storage conditions | Store at room temperature (1~30°C) |

* Other generic drugs (same ingredients, same dosage form) are also acceptable.

- 1. Subject/control drug management

The investigational drug used in this study will be purchased with research funds provided by the principal investigator and stored appropriately at each participating medical institution. The drug will be directly provided to participants for research purposes by the principal investigator, responsible investigator, or co-investigator. Any unused or expired drug due to missed doses will be disposed of according to the standard drug disposal procedures within the institution.

The control drug used in this study will be prescribed as part of routine clinical practice using standard prescriptions.

- 1. Selection Policy for Research Subjects

Eligible participants are those who meet all the inclusion criteria and none of the exclusion criteria as outlined below.

#### Inclusion Criteria:

- - 1. Patients undergoing pharmacotherapy for solid cancers, excluding head and neck cancers and hematological cancers, at participating facilities. The type or combination of anticancer agents, molecular targeted drugs, or immune checkpoint inhibitors does not matter.
    2. Patients aged 18 years or older at the time of consent.
    3. Patients who have received a thorough explanation of the study, fully understand its purpose and procedures, and have provided written informed consent voluntarily.

#### Rationale for Inclusion Criteria:

1. To ensure the study targets the intended population.
2. To include patients capable of making informed decisions.
3. To comply with the spirit of the Declaration of Helsinki.

#### Exclusion Criteria:

1. Patients with a history of hypersensitivity to betamethasone sodium phosphate.
2. Patients unable to perform gargling.
3. Patients with existing oral mucositis.
4. Patients diagnosed with oral candidiasis.
5. Patients deemed unsuitable for the study by the principal investigator, responsible investigator, or co-investigator.

#### Rationale for Exclusion Criteria:

1. To ensure the safety of study participants.
2. To include only those for whom the study can be conducted.
3. Since this study focuses on the preventive effects of the drug, patients with existing oral mucositis are excluded.
4. To avoid the potential risk of exacerbating oral candidiasis through the use of steroid gargles.

To ensure both participant safety and proper study implementation by allowing investigators to exclude unsuitable candidates.

- 1. Overview of treatment (protocol treatment)

Patients undergoing pharmacotherapy for solid cancers, excluding hematological cancers and head and neck cancers, will be divided into two groups for evaluation. After obtaining consent and completing registration, participants will be randomly assigned to either the betamethasone gargle group or the azulene gargle group. The evaluation will include the items specified in section 4.10.

No oral mucositis

No development of oral candidiasis

Betamethasone

8 weeks follow-up

Allocarion

Registratiton

Aznol containment

8 weeks follow-up

After confirming that eligible patients meet the inclusion criteria and do not have oral mucositis or oral candidiasis, they will be registered and randomly assigned to either the intervention group or the control group in a 1:1 ratio.

Intervention Group: In addition to standard oral care*, patients will gargle with 10 ml of 0.01% betamethasone solution four times daily (after each meal** and before bedtime), swishing it in their mouths for 30 seconds to 1 minute.

Control Group: In addition to standard oral care*, patients will gargle with 10 ml of 4% azulene solution four times daily (after each meal** and before bedtime), swishing it in their mouths for 30 seconds to 1 minute.

The use of steroid ointments on the oral mucosa is prohibited for both groups.

Note: For patients who do not eat meals, the gargle should be performed at meal-equivalent times. Standard Oral Care:

Includes modifications to dietary consistency, administration of analgesics or opioids, oral hygiene measures, and use of moisturizers.

Method for Diagnosing Oral Candidiasis:

Oral candidiasis is diagnosed if symptoms suggestive of candidiasis are identified by the attending cancer physician or oral care specialist, irrespective of whether a culture test is performed or its results.

- 1. Criteria for treatment (protocol treatment)

If oral candidiasis occurs during the treatment, gargling will be suspended in the betamethasone group and resumed once the candidiasis resolves. In the azulene group, gargling will continue even if oral candidiasis is detected.

- 1. Concomitant treatment

No specific concurrent drugs or therapies are mandatory for this study.

- 1. Prohibited Drugs / Devices / Therapies

The use of steroid ointments on the oral mucosa is prohibited during the study period.

- 1. Combined use of limiting shalf/combined use of limiting machine/combined use of limiting therapy

There are no restricted drugs or therapies defined for this study.

- 1. Assessments, collection items and schedules
- Schedule (Table)

|  | Regustration/assignment | Medical examination | | | | At exit | At the time of cancellation |
| --- | --- | --- | --- | --- | --- | --- | --- |
|  |  | 1~2 weeks | | | | Week 8 ends |  |
| Obtaining  consent | ● | － | － | － | － | － | － |
| Allocation | ● | － | － | － | － | － | － |
| Information gathering 1 | ● | － | － | － | － | － | － |
| Information gathering 2 | － | ● | ● | ● | ● | － | － |
| Information gathering 3 | － | － | － | － | － | ● | ● |

*1 The date of registration can be before or after the start of chemotherapy. The date of registration and the date of allocation can be different days.

*2 Once a week for inpatient treatment or at the time of hospitalization, and on the day of treatment for outpatient treatment

In the case of postponement of cool, inclusion and information gathering will be carried out according to the postponement

Dental examinations are not mandatory, and dentists are allowed to visit outpatient chemotherapy rooms for examinations

- Observation and Inspection Items

[Items to be collected at the time of registration]

| Item | Detail |
| --- | --- |
| Patient  Background | Age, sex, anamnesis and medication administered, primary site, smoking  history, alcohol consumption history, weight |
| Treatment-  related factors | The day of the start of treatment, the name of the anti-cancer drug, and the  use of drug/ combined therapy |
| Clinical laboratory findings (In-hospital  examination) | Neutrophil count, lymphocyte count, albumin, creatinine |
| Oral findings | Presence and grade of oral mucositis (CTCAE v3.0 and v5.0), number of remaining teeth, presence or absence of dentures, periodontal examination values (periodontal pockets, bleeding, upset), degree of plaque adhesion (OHI-S), degree of denture cleaning (in the case of dentures), presence and location of untreated teeth, degree of alveolar bone resorption in patients with dental X-rays |

[Information to be collected at the time of visit after allocation]

| Item | Detail |
| --- | --- |
| Oral mucositis | Presence and grade of oral mucositis (CTCAE v3.0 and v5.0), and date of onset in cases |
| Candida onset | Presence and timing of onset |
| Drugs for research use | Usage  Presence or absence of adverse events related to the study drug (inflammatory changes such as redness and swelling of the oral mucosa) |
| Adverse events | Presence or absence of the development of taste disorders, oral infections and other adverse events |

### [Information to be collected at the time of termination or cancellation]

| Item | Detail |
| --- | --- |
| Treatment-related factors | Chemotherapy start ~ end date, whether or not chemotherapy has been completed, use of analgesics, use of local anesthetics (sputum, jelly, etc.), use of opioids, neutrophil count (lowest during treatment), lymphocyte count (lowest during treatment), albumin (lowest value during treatment), creatinine (highest value during treatment), presence and timing of use of GCF-S preparations, PNI |
| Oral mucositis | Presence and grade of oral mucositis (CTCAE v3.0 and v5.0), and date of onset in cases |
| Candida onset | Presence and date of onset |
| Drugs for Research Use | Usage  Presence or absence of adverse events related to the study drug (inflammatory changes such as redness and swelling of the oral mucosa) |
| Adverse events | Presence or absence of the development of taste disorders, oral infections and other adverse events |

- 1. Treatment after the end of the study

The betamethasone gargle used in this study is not covered by insurance for the treatment of oral mucositis. Therefore, it will not be possible to continue the same treatment after the study concludes. After the study, the best alternative treatment will be selected from those available under insurance coverage, with the aim of minimizing any potential disadvantages.

- 1. Provision of test results, etc. to research subjects

As the study results do not directly impact the participants' subsequent treatment, the results will not be provided to the participants unless they request them.

- 1. Discontinuation criteria in individual study subjects [Discontinuation criteria]
- If the participant requests to discontinue their participation.
- If the occurrence of an adverse event (e.g., exacerbation of the primary disease, emergence of complications, development of a new disease) leads the investigator to determine that continuing the study is no longer appropriate for the participant.
- If, for any other reason, the investigator determines that continuing the study is not appropriate for the participant.

[Post-Discontinuation Procedures]

If discontinuation occurs, the use of the betamethasone gargle will be immediately stopped. The participant will then transition to a post-study observation period, with follow-up until the final visit whenever possible. Appropriate alternative treatments will be considered for each participant after discontinuation, and treatment will be adjusted accordingly.

# Predicted benefits and disadvantages for the study subject

- 1. Projected Profits

Participation in this study may reduce the incidence of oral mucositis caused by betamethasone. Additionally, the findings from this study could potentially contribute to the advancement of medical care in the future.

- 1. Foreseeable disadvantages

There are no direct risks associated with participation in this study; however, allergic reactions such as rashes or hypersensitivity to betamethasone may occur. Additionally, side effects such as the development of oral candidiasis or exacerbation of oral infections may occur. In case these side effects arise, gargling with betamethasone will be immediately discontinued, the participant will gargle with water, and antifungal or antibacterial treatments will be considered if necessary.

- 1. Expected side effects Hypersensitivity reactions to betamethasone Development of oral candidiasis Exacerbation of oral infections
  2. Expected side effects Not applicable

# Treatment if not participating in the present study

The investigational drug used in this study is not covered by insurance for the condition being treated. Therefore, if a participant does not join the study, treatment will be selected from available therapies covered by insurance and provided to the patient.

Potential treatments include azulene gargle or dexamethasone ointment.

When determining the treatment, the benefits and risks of the medical options will be explained to the patient before proceeding with the treatment.

| Treatment methods | Features and Efficacy | Typical side effects |
| --- | --- | --- |
| Aznol mouthwash | Anti-inflammatory effect | Hypersensitivity |
| Dexamethasone ointment | Symptomatic relief of stomatitis | Allergies, oral infections |

# Target number of cases

296 participants (148 participants in the betamethasone gargle group, 148 participants in the azulene gargle group)

[Rationale for setting]

Assuming an incidence rate of 40% for oral mucositis during chemotherapy for malignant tumors, a previous study by Kuba et al. showed that the risk of developing oral mucositis with steroid gargles is reduced to 0.68 times, i.e., 27%. Using two tailed alpha of 0.2, a power of 0.8, and accounting for a 10% dropout rate, the target sample size was set to 296 participants (148 in the intervention group and 148 in the control group).

# Duration of the study

Study Duration: From the JRCT publication date to December 31, 2026

Case Registration Period: From the JRCT publication date to March 31, 2026 Case Report Form Submission Deadline: June 30, 2026

# How to Register a Case

The principal investigator or co-investigator of the research site shall fill in the necessary items on the "Study Subject Screening List (Correspondence Table)" after obtaining written consent from the research subject.

The responsible investigator or co-investigator at the participating medical institution will obtain written informed consent from the participant and fill in the required information on the "Participant Screening List (Correspondence Table)."

The responsible investigator or co-investigator will confirm that the participant meets all inclusion criteria and none of the exclusion criteria, then complete the case registration form. The completed form will be emailed to the data management representative, , Kyushu Dental University.

・Patient registration address

・Name of institution: Kyushu Dental University, Department of Oral Health,

at Kyushu Dental University will review the case registration form. If the participant is deemed eligible, a registration confirmation letter with the registration number and allocation result will be issued. The confirmation letter will be emailed to the responsible investigator or co-investigator at the participating medical institution.

If the participant is deemed ineligible, will notify the responsible investigator or co- investigator with the reason for ineligibility.

The responsible investigator or co-investigator will verify the case registration confirmation letter received and initiate the betamethasone gargle or azulene gargle as indicated in the confirmation letter.

# Randomization Method and Allocation Adjustment Factors

Randomization Method: Participants will be randomly allocated in a 1:1 ratio to the betamethasone gargle group or azulene gargle group using computer software (stratified block randomization).

Allocation Factors: Presence of oral mucositis risk drugs (High risk: Drugs with an oral mucositis incidence rate of 30% or more as stated in the drug package insert).

Registration and Allocation Institution: Kyushu Dental University

# Managing Blinding

- 1. Methods of blinding Not applicable
  2. Determination and procedure for the need for key opening (key opening) Not applicable

## Evaluation items

- 1. Primary endpoint

## The presence of Grade 1 oral mucositis and the date of onset.

- 1. Secondary endpoints

Grade 2 and 3 oral mucositis onset and date of onset Oral candidiasis onset and date of onset

Completion of drug therapy

- 1. Safety endpoints

The presence of Grade 2 or 3 oral mucositis and the date of onset. The onset of oral candidiasis and the date of onset.

Whether the participant completed the pharmacotherapy.

# Statistical analysis

The analysis population and its definition are outlined below. The analysis population will be presented in a flowchart in accordance with the CONSORT statement.

| Population to be analyzed | Definition |
| --- | --- |
| Enrollment example  Intention To Treat (ITT) | All study subjects randomly assigned to the study |
| Population to be analyzed for safety analysis  Safety Analysis Set (SAS) | All study subjects for whom study medications were used |
| Largest Population Analyzed  Full Analysis Set (FAS) | Study subjects with ITT for whom a study drug was used and the primary endpoint was obtained |
| Target population that conforms to the research protocol  Per Protocol Set (PPS) | Study subjects with FAS who do not deviate from the research protocol. |

- 1. Identify the target population

【**Analysis Populations**】ITT,FAS,PPS

Summarize patient background using summary statistics.

| Population to be analyzed | Definition |
| --- | --- |
| Enrollment example  Intention To Treat (ITT) | All study subjects randomly assigned to the study |
| Largest Population Analyzed  Full Analysis Set (FAS) | Study subjects with ITT for whom a study drug was used and the primary endpoint was obtained |
| Target population that conforms to the research protocol  Per Protocol Set (PPS) | Study subjects with FAS who do not deviate from the research protocol. |

- 1. Primary endpoint analysis

【**Analysis Population**】FAS,PPS

Estimate the risk difference and risk ratio, along with their 95% confidence intervals, for the incidence of grade 1 oral mucositis between the betamethasone gargle group and the azulene gargle group. Based on the settings from the prior sample size calculation, a p-value < 0.2 derived from Fisher’s exact test will be considered statistically significant. The cumulative incidence at each time point will be estimated using the Kaplan–Meier method, and for reference, a log-rank test will be performed.

- 1. Analysis of secondary endpoints

【**Analysis Population**】FAS,PPS

1. Descriptive statistics will be used to summarize each evaluation criterion.
2. Estimate the risk difference and risk ratio, along with their 95% confidence intervals, for the incidence of grade 2 oral mucositis between the betamethasone gargle group and the azulene gargle group. Based on the settings from the prior sample size calculation, a p-value < 0.2 derived from Fisher’s exact test will be considered statistically significant. The cumulative incidence at each time point will be estimated using the Kaplan–Meier method, and for reference, a log-rank test will be performed. The incidence rate of oral candidiasis in the betamethasone gargle group and the azulene gargle group will be calculated using the Kaplan-Meier method, and the difference between the two groups will be analyzed using the log-rank test.
3. Factors associated with the incidence of oral mucositis for each grade will be analyzed using Cox regression analysis.
   1. Analysis of Safety Assessment

The adverse events in the betamethasone gargle group and the azulene gargle group will be described.

- 1. Subpopulation analysis Not applicable
  2. Interim Analysis Plan Not implemented
  3. Handling of missing data, etc.

In cases where some data points for investigation items are missing, those cases will not be discarded, and the data will still be included in the statistical analysis.

- 1. Changing the Statistical Analysis Plan

If any changes to the original statistical analysis plan are made during the study, the research protocol or statistical analysis plan will be revised, and the changes will be explained in the final study report.

# Data Management

- 1. Types of case report forms

For this study, paper-based case report forms (CRFs) will be used. The responsible investigator or co- investigators at the participating medical institutions will prepare a CRF for each participant. The CRF will contain the data required by the study protocol, and any changes or corrections will be made following the "Case Report Form Entry and Correction Guidelines" (to be attached if created, or referenced in the protocol if not created). When making changes or corrections, the responsible investigator or co-investigator will cross out the corrected portion with a double line, sign or stamp the correction, and indicate the date of the correction. If the correction pertains to an important matter as defined in the "Case Report Form Entry and Correction Guidelines," the reason for the correction will also be included along with the date.

The original CRF, signed by the responsible investigator, will be considered the official document, and the responsible investigator will guarantee that the recorded data is complete and accurate. During the observation period, copies of the CRFs will be submitted to Kyushu Dental University. If there are any changes or corrections, they will be made on the original CRF, and the corrected copy will be resubmitted.

After the observation period, the responsible investigator will prepare copies of all CRFs and submit the original CRFs by mail or in person to Kyushu Dental University. Copies of the CRFs will be kept securely at each participating institution for the duration specified in the study protocol.

- 1. Identification of source materials

The source documents for this study are as follows:

- Records related to the participant's consent and information provided to the participant
- Medical records, image films, and other records used to create the case report form
- Other records related to the implementation of this study kept by the participating medical institutions
  1. Items that are entered directly in the case report form and should be interpreted as source materials

For this study, the following items will be considered as source data in the case report form:

- For clinical laboratory values, if values fall outside of the reference range but are not clinically problematic, the judgment and reason for this decision will be recorded.
- For adverse events, the reason for determining that follow-up is not required will be recorded.
- The reasons for using concomitant medications or treatments will be recorded.

# Handling of Diseases, etc.

- 1. Definition of terms Adverse Events:

Any unfavorable or unintended signs (including abnormal clinical research values), symptoms, or diseases that occur during the study period, regardless of whether there is a causal relationship with the conduct of the specific clinical study.

Diseases, etc.:

Any diseases, disorders, deaths, infections, abnormal clinical laboratory values, or other symptoms that are suspected to be caused by the implementation of the specific clinical study. In this study, any events suspected to be caused by the study will be handled as diseases or adverse events.

- 1. Evaluation of diseases, etc.

The causal relationship between any adverse event and the study will be determined by the principal investigator, responsible investigator, or co-investigator. This determination will consider the temporal relationship with the intervention, the course of the underlying disease, complications, concomitant medications, study procedures, accidents, and other external factors. The causal relationship will be evaluated and recorded according to the following criteria:

- Causal Relationship Present or Cannot Be Excluded:
  - It is reasonable or likely that the study or intervention caused the event.
  - A temporal relationship exists between the study and the event.
  - No other cause can be identified, and the causal relationship with the study cannot be excluded.
- No Causal Relationship:
  - It is not reasonable to attribute the event to the study or intervention.
  - No temporal relationship can be demonstrated.
  - Another cause can be identified.
  1. Predictable diseases, etc. Development of oral candidiasis Exacerbation of oral infections

Other side effects listed in the package insert for the investigational drug

- 1. Measures to be taken for research subjects in the event of illness, etc.

If any diseases or adverse events occur, the responsible investigator or co-investigator will promptly take appropriate actions (including explanations) and implement necessary measures such as discontinuing or interrupting the study. If any diseases or adverse events persist after the final observation, the participant will be followed until recovery to the baseline state or clinical stabilization.

- 1. Reporting of illnesses, etc.

If any diseases or adverse events occur, the responsible investigator or co-investigator will record the name of the event (diagnosis), date of occurrence, severity, causal relationship, predictability, outcome, and the date of the outcome assessment.

- 1. Reporting of serious illnesses, etc.

If any serious diseases or adverse events occur, the responsible investigator must promptly report them within the specified time frame to the medical institution's administrator and the Nagasaki University Clinical Research Review Board. For unpredictable diseases or adverse events, reports must also be submitted to the Ministry of Health, Labour and Welfare (PMDA) within the designated period.

| Unapproved/unapproved use submission deadline | | | | | |
| --- | --- | --- | --- | --- | --- |
|  | Type of adverse | Causality | Predictability | Committee report deadline | MMHL report deadline |
| ① | 1.death | yes | No | 7days | 7days |
|  | or threat of death |  | Yes | 15days | ‐ |
|  |  | No | ‐ | ‐ |  |
| ② | 2. hospitalization for treatment or prolonged | yes | No | 15days | 15days |
|  | 3. disability or threat of disability |  | Yes | ‐ | ‐ |
|  | 4. death or seriousness as 2-4 | No | ‐ |  |  |
|  | 5. later generation Congenital disease, etc.or abnormality in |  |  |  |  |

- 1. Reporting Defects Not applicable

# Effectiveness and Safety Evaluation Committee

An efficacy and safety evaluation committee will not be established for this study.

# Compliance, modification and non-conformity with the research plan (deviation from the research plan, etc.)

- 1. Compliance with the research protocol

The responsible investigator or co-investigator at the participating medical institution must obtain prior written approval from the clinical research review board, as outlined in the study protocol, and may not deviate from or modify the protocol without this prior approval.

- 1. Changes to the Research Proposal

If any changes to the study protocol or other aspects related to the study occur, the principal investigator will consult with the clinical research review board for their opinion and obtain approval from the administrator of the participating medical institution.

Additionally, if any changes occur in the implementation plan (including attachments), the principal investigator will update the JRCT information and submit the revised implementation plan to the Ministry of Health, Labour and Welfare.

- 1. Management of Non-compliance (Deviations from the Study Protocol)
     1. If the responsible investigator or co-investigator at a participating medical institution becomes aware of any non-compliance with the study, they must promptly report it to the administrator of the participating institution and notify the principal investigator.
     2. If any serious non-compliance is identified that could affect the rights, safety, progress, or reliability of the study, the principal investigator will consult the clinical research review board for their opinion.
     3. Serious non-compliance does not include deviations from the protocol made to avoid immediate risks to participants or due to unavoidable medical reasons.

# About Informed Consent

- 1. Procedure for obtaining informed consent

The responsible investigator or co-investigator at the participating medical institution will explain the study to the participant using an informed consent document that has been approved by the clinical research review board, as outlined in the implementation plan. After confirming that the participant fully understands the study, written consent will be obtained. A copy of the informed consent form and explanation document will be promptly provided to the participant.

The items that must be explained to the participant are specified in Article 46 of the Clinical Research Act and its enforcement regulations, under "Explanation and Consent for Participants in Specified

Clinical Studies" (refer to the consent form). However, any items recognized by the clinical research review board and approved by the institution’s administrator may be excluded from this requirement. If any information is received that could influence the participant's decision to continue in the study, the responsible investigator must promptly provide the participant with this information, record it, and verify whether the participant wishes to continue. If the informed consent document is revised, the responsible investigator or co-investigator must explain the revisions to the participant and obtain updated written consent for continued participation.

- 1. Handling of Inquiries from Participants or Their Relatives

Inquiries from participants or their relatives will be handled by the responsible investigator, co- investigator, or the study administration office.

- 1. When obtaining informed consent from a substitute, etc. Not applicable
  2. Informed Ascent Not applicable
  3. Cases Where Informed Consent Is Not Required Not applicable

# Handling of Personal Information, etc.

- 1. Management of Personal Information

In this study, each participant will be assigned an identification code. This code, consisting of non- identifiable numbers or symbols, will be used in all study-related documents (such as case report forms and case registration forms) to anonymize the data. The responsible investigator will create a correspondence table containing the participant's identifiable information (e.g., name, medical record ID) and will keep it securely stored to prevent unauthorized access. All individuals involved in the study will comply with applicable laws and regulations regarding the protection of personal information.

Personal information and privacy will be protected to the highest standard, and no personal information obtained during the study will be disclosed without legitimate reasons. This protection extends even after individuals leave the study. As this is a multicenter study, each participating institution will manage the correspondence tables appropriately, and no data will be provided outside the institutions.

When study-related information (such as case registration forms, case report forms, and test data) isprovided to other research institutions, only anonymized data will be shared (using the participant identification code or registration number, so that the participant cannot be immediately identified).In addition, the parties concerned shall make every effort to protect the personal information and privacy of the subjects, and shall not divulge personal information obtained in the course of conducting this research without a justifiable reason. The same shall apply even after the person concerned has retired from the position.

This study is a multicenter collaborative study, and the correspondence table will be appropriately managed at each medical institution, and will not be provided outside the participating medical institution.

When providing a study subject's case registration form, case report form, test data, etc. outside the research institution, anonymize it using the research subject identification code or registration number (limited to those that have been processed or controlled so that it is not immediately possible to determine which research subject's sample or information is the data or information). Personal information in this research will be handled in compliance with this research plan and procedures for the handling of personal information related to clinical research at each implementing medical institution.

- 1. Disclosure of retained personal information that can be used to identify the individual

If the research-responsible physician receives a request from an individual or their representative to disclose personal information that can identify the individual, they must promptly disclose the relevant personal information to the requester. However, disclosure may be partially or fully withheld if it falls under any of the following circumstances:

1. If disclosure is likely to harm the life, body, property, or other rights and interests of the individual or a third party.
2. If disclosure is likely to significantly hinder the proper conduct of the clinical research.
3. If disclosure would result in a violation of other laws or regulations.
   1. Handling of information on genetic characteristics, etc. (including accidental findings)

This study does not involve any testing or analysis that would reveal the genetic characteristics of participants. Therefore, this section does not apply.

- 1. Access to source documents.

The principal investigator and the participating medical institutions will provide access to all relevant clinical study records, including source documents, during monitoring, audits, or investigations by the clinical research review board and regulatory authorities.

# Storage and disposal of information

Biological samples will not be stored in this study. The principal investigator will strictly manage and store the documents and records listed below in a lockable storage cabinet.

For electronic data, it will be stored with password protection on devices such as standalone computers or USB drives that are isolated from the hospital LAN and the internet. When not in use, these devices will also be stored in a lockable storage cabinet for secure management.

The retention period for the documents and records is five years from the date the study concludes. Upon expiration of the retention period, documents and records will be disposed of with the utmost care to prevent the leakage of personal or confidential information. Paper documents will be shredded, while other media will be anonymized and deleted or disposed of using appropriate methods.

The research-responsible physician at the implementing medical institution will retain the following documents for five years after the study concludes, in accordance with the institution’s regulations:

1. Documents identifying study participants (correspondence table)*
2. Information regarding medical treatment and tests conducted for study participants
3. Matters related to participation in the specific clinical study (original case registration forms)
4. Study protocol
5. Implementation plan
6. Information sheets, consent forms, and withdrawal of consent forms
7. Signed consent forms (original copies)*
8. Case report forms (original copies)
9. Documents submitted for obtaining approval from the institution's administrator and the approval certificate
10. Documents related to monitoring and auditing (if audits are conducted)
11. Contracts related to the implementation of the specific clinical study (copies), excluding contracts with pharmaceutical manufacturers or their affiliates
12. Other documents or records related to this study (*) Indicates items containing identifiable information.

# Secondary use of information

There are no plans to use the samples or data obtained in this study for purposes other than the research itself. However, the data obtained in this study may be deposited in the UMIN-ICDR (<https://www.umin.ac.jp/icdr/index-j.html>).

# Use as a biobank of information

Not applicable

# Sources of funding and conflict of interest management

- 1. Sources of funding for the research, etc.

This research will be supported by a research grant from the Grant-in-Aid for Scientific Research (Grant-in-Aid for Scientific Research C, Reiwa 5~7) (Grant No. 23K09315).

- 1. Managing Conflicts of Interest

The principal investigator will establish a conflict of interest (COI) management policy for this study. The principal investigator will confirm the details of any COI disclosures related to the involvement of companies in the clinical research and will prepare a report on the relevant companies.

The individuals who have disclosed a conflict of interest must verify the facts with the administrator of the participating medical institution and obtain a COI confirmation report. Based on the COI confirmation report, the principal investigator will develop a conflict of interest management plan, consult with the clinical research review board, and implement appropriate management measures.

# Participant Costs and Compensation

Regarding the costs of this study, participants will be responsible for paying their own share of medical expenses (such as consultation fees, hospitalization fees, medication costs, and test fees), as all treatments in this study are covered under insurance. There will be no additional financial burden for participants from participating in the study. No monetary or non-monetary compensation will be provided to participants for their participation or any associated costs.

# Compensation for health hazards

Severe adverse events are considered very rare in this study, so clinical research liability insurance will not be purchased.

If any health damage occurs due to the implementation of this study, the responsible investigator or co-investigator will provide appropriate treatment and care.

Treatment will generally be covered by the participant’s health insurance, and any out-of-pocket expenses, such as for medical costs, sick leave compensation, or differential bed charges, will not be reimbursed.

# Periodic Reporting

- 1. Periodic reporting to accredited clinical research review boards/administrators

The principal investigator shall report the status of the implementation of the specified clinical research to the administrator of his or her institution every one year (within two months after the expiration of the period) from the date of submission of the implementation plan to the Minister of Health, Labour and Welfare, and shall make periodic reports to the Accredited Clinical Research Review Board described in the implementation plan. The matters to be reported shall be as follows:

1. Number of clinical research participants
2. Occurrence of diseases and subsequent course
3. Occurrence of non-conformity and subsequent response
4. Evaluation of safety and scientific validity
5. Matters related to the involvement of pharmaceutical manufacturers, distributors, etc., as stipulated in the Conflict of Interest Management Standards

In addition, the principal investigator must promptly report to the administrator of the implementing medical institution after reporting to the Accredited Clinical Research Review Board.

When the Principal Investigator reports to the Accredited Clinical Research Review Board, the Principal Investigator shall promptly provide information to the other Principal Investigators. The other investigator shall promptly report the content of the provision of such information to the administrator of each medical institution.

### Periodic Report to the Minister of Health, Labour and Welfare

The principal investigator will report to the Minister of Health, Labour and Welfare within one month of receiving an opinion from the clinical research review board regarding the continuation of the study. The report will include:

1. The name of the committee listed in the implementation plan
2. Appropriateness of the Committee's continuation of the specific clinical research
3. Number of Specified Clinical Research Subjects Participating in Specified Clinical Research

# Termination and Discontinuation of the Study

- 1. Termination of study

See 28.2 Publication of research results.

- 1. Discontinuation of the study Discontinuation Criteria

The study may be discontinued if the following situations occur:

- Unexpected severe diseases or events that could adversely affect the participants
- Significant violations of laws, regulations, or the study protocol
- Facts that undermine the ethical or scientific validity of the study
- Identification of significant risks to participants
- An opinion from the clinical research review board to discontinue the study
- A request or recommendation for discontinuation from the Minister of Health, Labour and Welfare

Discontinuation Procedures

If the study is discontinued, the principal investigator must notify the clinical research review board within 10 days and submit a discontinuation notice to the Minister of Health, Labour and Welfare. The principal investigator will consult with the clinical research review board as necessary.

After submitting the discontinuation notice to the Ministry, regular progress reports will continue to be submitted, and if any changes occur, the implementation plan will be updated.

Participant Handling in Case of Discontinuation

The responsible investigator will contact participants to inform them of the study discontinuation, explain the next steps, and discuss the follow-up schedule. When discontinuation occurs, ethical considerations and participant safety will be carefully reviewed to minimize risks.

# Disclosure of research information and results

- 1. Enrollment of studies

Prior to the implementation of this study, it will be recorded (registered) in a database (j RCT = Japan Registry of Clinical Trials) maintained by the Ministry of Health, Labour and Welfare.

The research plan will be updated as appropriate according to changes and progress of the research, and the results of the research will be registered when the research is completed.

- 1. Publication of research results

The principal investigator must prepare a final report and its summary within one year after the end of the period for collecting data related to all evaluation items.

(As a general rule, the final report must be prepared and submitted within one year from the later of the date the study was discontinued or the date the data collection period for all evaluation items ended.)

Once the final report and its summary are prepared, the principal investigator must seek the opinion of the Certified Clinical Research Review Board (CCRRB). Within one month of the CCRRB providing its opinion, the principal investigator must submit the final report to the administrator of the implementing medical institution and the summary of the report to the Minister of Health, Labour and Welfare, and publish it on the jRCT (Japan Registry of Clinical Trials) system.

The date when the summary of the final report is published on jRCT will be considered the study’s completion date. Upon publication in jRCT, the principal investigator must promptly notify the administrator of the implementing medical institution of the publication.

When submitting the final report to the administrator of the implementing medical institution, the principal investigator must also notify other research-responsible physicians. Those physicians must promptly report the provided information to the administrators of their respective implementing medical institutions. Similarly, when the summary of the final report is published on jRCT, the principal investigator and research-responsible physicians must promptly share this information as well.

- 1. Sharing anonymized data of individual subjects of specific clinical studies

If another researcher makes a request based on a reasonable rationale, there is a possibility that anonymized data from individual participants in the specific clinical research may be shared with the requesting researcher.

# Quality Control & Quality Assurance

- 1. monitoring

The principal investigator shall appoint a monitoring officer to conduct monitoring to ensure the following:

The rights and safety of study participants are protected.

The study is conducted in compliance with the implementation plan, the study protocol, and applicable regulations.

The reliability of the data is maintained.

Matters related to the conduct of monitoring will be specified in a separately prepared "Monitoring Procedures (Plan)." The monitoring officer shall perform monitoring in accordance with the provisions of the "Monitoring Procedures (Plan)."

- 1. audit

In this study, no audits were conducted.

# Attribution of Research Results (Intellectual Property Rights)

There is a possibility that patent rights and economic benefits may arise from this research, but the rights belong to the research institute or the principal investigator who conducts the research, and the research subjects do not have this right.

In addition, the research results conducted based on this research plan shall be those of Nagasaki University.

# Research Implementation Structure


Affiliation: Nagasaki University Hospital

- 1. Research Secretariat

Not established.

- 1. Participating Medical Institutions and Responsible Investigator

Refer to Implementation Plan (Form No. 1) 1(4)

- 1. Data Management Officer Name:

Affiliation: Kyushu University of Dental Sciences

- 1. Statistical Analysis Officer:

None

- 1. Monitoring Officer

Affiliation: Nagasaki University Hospital

- 1. Audit Officer

None

- 1. R&D Planning Support Officer

None

- 1. Coordination and administrative staff

None

- 1. A person who supervises the research other than the principal investigator or the principal investigator

Affiliation: Aichi Gakuin University

- 1. Other clinical laboratories related to clinical research, as well as medical and technical departments and institutions

None

- 1. Contract Development Organizations

None

## References

1. Lalla RV, et al. MASCC/ISOO clinical practice guidelines for the management of mucositis secondary to cancer therapyCancer, 120: 1453-1461, 2014.
2. Tadashi Ueno, et al. Results of a double-blind clinical trial of dexartin ointment.Journal of Japan, 26:1399-1408,1980.
3. Teshima, A., et al. A study on the efficacy and safety of Kenalog ointment for radiation stomatitis in patients with head and neck tumors.Pharmacology and Treatment, 14:7163-7166,1986.
4. Textbook of the National Common Cancer Medical and Dental Collaborative Workshop, 2nd Edition, 2018.
5. Nshii M, et al. Factors associated with severe oral mucositis and candidiasis in patients undergoing radiotherapy for oral and oropharyngeal carcinomas: a retrospective multicenter study of 326 patients. Support Care Cancer, 28(3): 1069-1075, 2020.
6. Baselga J, et al. Everolimus in postmenopausal hormone-receptor-positive advanced breast cancer. N Engl J Med,366: 520-529, 2012.
7. Rugo HS, et al. Prevention of everolimus-related stomatitis in women with hormone receptor- positive, HER2-negative metastatic breast cancer using dexamethasone mouthwash (SWISH): a single-arm, phase 2 trialLancet Oncol, 18: 654-662, 2017.
8. Niikura, et al. Oral Care Evaluation to Prevent Oral Mucositis in Estrogen Receptor-Positive MetastaticBreast Cancer Patients Treated with Everolimus (Oral Care-BC): A Randomized Controlled Phase III Trial. Oncologist, 25(2):223-e230 2020.
9. Umeda, et al. Oral care and oral assessment guide in breast cancer patients receiving everolimus and exemestane: subanalysis of a randomized controlled trial (Oral Care-BC). Ann Transl Med, 9(7): 535 2021.
10. Kuba S, et al. Efficacy and safety of a dexamethasone-based mouthwash to prevent chemotherapy-induced stomatitis in women with breast cancer: A multicenter, open-label, randomized phase 2 study. J Evid-Based Dent Pract, 23:101896. 2023.
11. Fernández-Sala X, et al. Effectiveness and safety of a novel dexamethasone mouthwash formulation in managing stomatitis in cancer patients. Farm Hosp. 45:41-44, 2020．
