# Supplementary material for: Prevention of oral mucositis in patients undergoing cancer chemotherapy using betamethasone mouthwash: A multicenter randomized controlled trial protocol
Source: PLoS One. 2026 Apr 8;21(4):e0345991. doi: 10.1371/journal.pone.0345991 (PMC13061232; doi:10.1371/journal.pone.0345991)
Supplement: S4 File — (DOCX) [file pone.0345991.s004.docx]

Supporting Information

S5: Membership of the Research Committee of the Japanese Society of Oral Care. The members of the Research Committee of the Japanese Society of Oral Care are as follows:

Masahiro Umeda：Aichi Gakuin University

Takaaki Ueno：Osaka Medical and Pharmaceutical University

Mitsunobu Otsuru：Kanagawa Dental University

Hiroshi Kurita：Shinshu University

Daichi Chikazu：Tokyo Medical University

Hitoshi Kawamata：Dokkyo Medical University

Yasuyuki Shibuya：Nagoya City University

Akira Tanaka：Nippon Dental University School of Life Dentistry at Niigata

Hideto Imura：Nagasaki University

Sakiko Soutome：University of Toyama

Yumiko Ikegami　Baika Women’s University

Toshiro Yamamoto：Kyoto Prefectural University of Medicine

Teruyuki Niimi：Aichi Gakuin University
